# Enumeration of Rooted Binary Unlabeled Galled Trees

**DOI:** 10.1007/s11538-024-01270-8

**Published:** 2024-03-22

**Authors:** Lily Agranat-Tamir, Shaili Mathur, Noah A. Rosenberg

**Affiliations:** Department of Biology, Stanford University, Stanford, CA 94305 USA

**Keywords:** Galled trees, Phylogenetics, Unlabeled trees

## Abstract

Rooted binary *galled* trees generalize rooted binary trees to allow a restricted class of cycles, known as *galls*. We build upon the Wedderburn-Etherington enumeration of rooted binary unlabeled trees with *n* leaves to enumerate rooted binary unlabeled galled trees with *n* leaves, also enumerating rooted binary unlabeled galled trees with *n* leaves and *g* galls, $0 \leqslant g \leqslant \lfloor \frac{n-1}{2} \rfloor$. The enumerations rely on a recursive decomposition that considers subtrees descended from the nodes of a gall, adopting a restriction on galls that amounts to considering only the rooted binary *normal* unlabeled galled trees in our enumeration. We write an implicit expression for the generating function encoding the numbers of trees for all *n*. We show that the number of rooted binary unlabeled galled trees grows with $0.0779(4.8230^n)n^{-\frac{3}{2}}$, exceeding the growth $0.3188(2.4833^n)n^{-\frac{3}{2}}$ of the number of rooted binary unlabeled trees without galls. However, the growth of the number of galled trees with only one gall has the same exponential order 2.4833 as the number with no galls, exceeding it only in the subexponential term, $0.3910n^{\frac{1}{2}}$ compared to $0.3188n^{-\frac{3}{2}}$. For a fixed number of leaves *n*, the number of galls *g* that produces the largest number of rooted binary unlabeled galled trees lies intermediate between the minimum of $g=0$ and the maximum of $g=\lfloor \frac{n-1}{2} \rfloor$. We discuss implications in mathematical phylogenetics.

## Introduction

Evolutionary histories of genes, populations, and species are often described by phylogenetic trees that seek to represent their descent relationships. Owing in part to the centrality of phylogenetic trees in evolutionary biology, mathematical studies have characterized numerous classes of phylogenetic trees, investigating their combinatorial properties (Semple and Steel 2003; Felsenstein 2004; Gascuel 2005; Steel 2016; Warnow 2018).

The use of tree structures—typically treated as binary—is often appropriate for representing standard phenomena of evolutionary descent, by which biological entities sequentially bifurcate, in a manner in which diverged entities do not merge back together. Processes such as genetic admixture, horizontal gene transfer, and hybridization, however, produce evolutionary relationships that are not tree-like. These processes involve the merging of separate lineages that had previously descended from shared ancestors. With increasing interest in merging mechanisms during evolutionary descent, much recent attention in mathematical phylogenetics has been devoted to phylogenetic networks (Huson et al. 2010; Gusfield 2014; Kong et al. 2022), in which graphs describing relationships of biological entities permit certain types of cycles.

Among the simplest phylogenetic networks are the *galled trees*, named for the growths that can appear in plant tissues to produce distinctively shaped structures (Gusfield et al. 2003, 2004b). First introduced in studies of ancestral recombination graphs (Wang et al. 2001; Gusfield et al. 2003, 2004a, b; Gusfield 2005, 2014; Song 2006), galled trees allow diverged lineages to merge forward in time, but only in circumscribed ways. Each merging event creates a *gall* corresponding to a cycle in the associated network.

From a standpoint that considers galled trees as mathematical objects separately from the processes that could produce them biologically, the defining feature of a galled tree is that cycles in a graph structure are disjoint, so that in a galled tree, a vertex or edge is contained in at most one cycle (Semple and Steel 2006). With this graph-theoretic sense for the meaning of galled trees, the enumerative combinatorics of galled trees has been investigated, both for unrooted and for rooted binary galled trees, focusing on galled trees that are leaf-labeled (Semple and Steel 2006; Bouvel et al. 2020; Cardona and Zhang 2020).

Chang et al. (2018) and Mathur and Rosenberg (2023) have posed the problem of enumerating rooted binary galled trees in which the leaves are *not* labeled. In a study focused on introducing encodings for galled trees, Chang et al. (2018) argued that the number of rooted binary unlabeled galled trees with *n* leaves is bounded above by a sequence with a certain generating function. In an enumerative study of labeled histories for rooted binary leaf-labeled galled trees, Mathur and Rosenberg (2023) enumerated a class of rooted binary unlabeled galled trees for *n* from 1 to 6, obtaining 1, 1, 2, 6, 20, 72. These values are indeed bounded above by the corresponding upper bounds of Chang et al. (2018)—1, 1, 4, 28, 245, 2402 for $n=1$ to 6—though Chang et al. (2018) used a more expansive definition of rooted binary unlabeled galled trees. They are also bounded above by the enumeration in Theorem 8 of Cardona and Zhang (2020) of the corresponding set of rooted binary *labeled* galled trees, which gives 1, 1, 6, 69, 960, 24,750 for $n=1$ to 6.

How many rooted binary unlabeled galled trees possess a given number of leaves *n* and a given number of galls *g*? Here, we perform this general enumeration, counting the rooted binary unlabeled galled trees of Mathur and Rosenberg (2023) for $n \geqslant 1$ leaves and $g \geqslant 0$ galls. We first recursively enumerate all such rooted binary unlabeled galled trees with a specified number of leaves *n*, considering all possible numbers of galls. We then refine this enumeration by subdividing it according to specified numbers of leaves *n* and galls *g*, considering all possible values of *g* for a fixed *n*.

## Background

### Definitions

We follow Mathur and Rosenberg (2023) in describing key concepts, assuming that all networks and trees are binary (and henceforth dropping the term *binary*). A *rooted phylogenetic network* is a directed acyclic graph with four properties: (i) there exists a unique *root node* with in-degree 0 and out-degree 2; (ii) all *leaf nodes* have in-degree 1 and out-degree 0; (iii) all non-leaf, non-root nodes have in-degree 2 and out-degree 1 or in-degree 1 and out-degree 2; and (iv) all edges are directed away from the root. Nodes with in-degree 2 and out-degree 1 are termed *reticulation nodes*, and nodes with in-degree 1 and out-degree 2 are *tree nodes*.

**Fig. 1:**
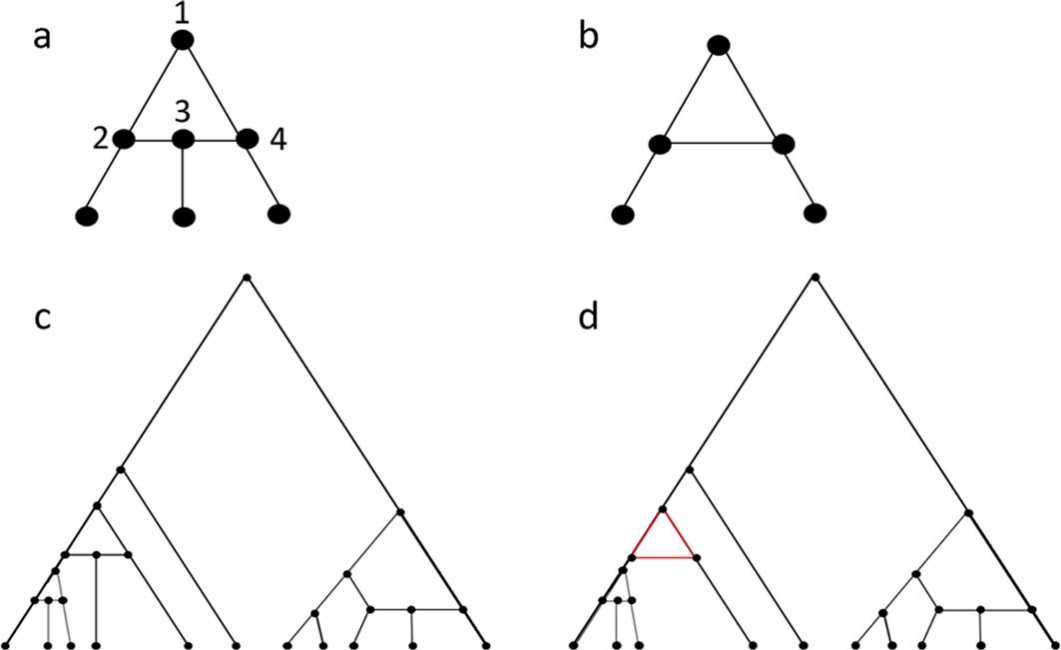
Rooted galled trees. **A** A rooted galled tree. In our definition of rooted galled trees, this example is the smallest network that possesses a gall. The gall is a root gall; node 1 is *r*, the *top node*; node 2 is the *left hybridizing side node*; node 4 is the *right hybridizing side node*; finally, node 3 is $a_r$, the *hybrid node*. We depict the hybridizing side nodes and the hybrid node in a horizontal line, representing simultaneity of these nodes in the embedding of the rooted galled tree in time, proceeding from the top to the bottom of the diagram. **B** A network that does not satisfy our definition of a rooted galled tree, but that does qualify according to some definitions. This network is missing a hybrid node; each gall in our definition possesses at least four nodes. **C** A more complex rooted galled tree by our definition. **D** A more complex network that is not a rooted galled tree by our definition, because the red triangle lacks a hybrid node

A *rooted galled tree* is a rooted (binary) phylogenetic network in which two properties hold (Fig. 1). First, (i) each reticulation node $a_r$ has a unique ancestor node *r* such that exactly two non-overlapping paths of edges exist from *r* to $a_r$; if the direction of edges is ignored, then the two paths connecting *r* and $a_r$ form a cycle $C_r$, known as a *gall*. Following Mathur and Rosenberg (2023), the ancestor node *r* must be separated from $a_r$ by at least two edges. This requirement that cycles contain at least four nodes is required by the perspective of Mathur and Rosenberg (2023) that views galled trees as evolving temporally by a biological process such as hybridization. It is equivalent to the requirement that a galled tree be a *normal network* and is not imposed in a more expansive galled tree definition that permits 3-node galls (Kong et al. 2022).

The second criterion is: (ii) the set of nodes in the gall $C_r$, associated with reticulation node $a_r$, and the set of nodes in the gall $C_s$, associated with reticulation node $a_s \ne a_r$, are disjoint.

We term the ancestor node *r* of a gall with reticulation node $a_r$ and cycle $C_r$ the *top node*. Other nodes in a gall, excluding the top node and reticulation node, are called *side nodes*. We term the reticulation node a *hybrid node*, and the two immediate parents of a hybrid node *hybridizing side nodes*, or just *hybridizing nodes*. The side nodes to the left and right of the hybrid node are the *left side nodes* and *right side nodes*, respectively; the distinction between “left” and “right” is only for convenience, and a gall is invariant with respect to exchange of its left and right side nodes. If the root node of a rooted galled tree is part of a gall, then we call this gall the *root gall*. The root node is always a top node if it is part of a gall.

Although a rooted galled tree is only strictly a tree if it contains no galls, it is convenient to continue to refer to galled trees as trees; similarly, we allow “subtrees” to possess galls. All networks and trees that we consider are rooted, and we henceforth drop the term *rooted*. Mathur and Rosenberg (2023) focused on *labeled* galled trees, in which each leaf is associated with a distinct leaf label; here we consider *unlabeled* galled trees, and we often drop the term *unlabeled*. Unlike Mathur and Rosenberg (2023), we have no need to assign a temporal embedding to nodes, with the exception that ancestor nodes can be no more recent than their descendants; the galled trees that we consider are understood to be *unordered*.

### Compositions

We will have occasion to consider the sums of ordered *b*-tuples of positive integers that equal a positive integer *a*: the compositions *C*(*a*, *b*) of *a* into *b* parts, $1 \leqslant b \leqslant a$. The set *C*(*a*, *b*) has cardinality $$|C(a,b) |=\left( {\begin{array}{c}a-1\\ b-1\end{array}}\right) $$. This result is obtained by noting that a list of *a* copies of the number 1 has $a-1$ “breakpoints” between consecutive 1’s, and, summing 1’s between neighboring breakpoints, the compositions into *b* parts are produced by the distinct sets of $b-1$ among the $a-1$ breakpoint locations. For $b>a$, we define $C(a,b)=\emptyset$, with $|C(a,b) |= 0$.

**Table 1:** The number of palindromic compositions of *a* into *b* parts, $1 \leqslant b \leqslant a$, and the corresponding number of non-palindromic compositions

Parity of *a*	Parity of *b*	$|C_p(a,b)|$	$|C_{np}(a,b)|$
Even	Even	${\frac{a}{2}-1 \atopwithdelims ()\frac{b}{2}-1}$	${a-1 \atopwithdelims ()b-1} - {\frac{a}{2}-1 \atopwithdelims ()\frac{b}{2}-1}$
Even	Odd	${\frac{a}{2}-1 \atopwithdelims ()\frac{b-1}{2}}$	${a-1 \atopwithdelims ()b-1} - {\frac{a}{2}-1 \atopwithdelims ()\frac{b-1}{2}}$
Odd	Even	0	${a-1 \atopwithdelims ()b-1}$
Odd	Odd	${\frac{a-1}{2} \atopwithdelims ()\frac{b-1}{2}}$	${a-1 \atopwithdelims ()b-1} - {\frac{a-1}{2} \atopwithdelims ()\frac{b-1}{2}}$

We distinguish between *palindromic* and *non-palindromic* compositions. Palindromic compositions are unchanged when the order of the parts is reversed; non-palindromic compositions do change when the order is reversed. For example, in *C*(9, 5), (1, 2, 3, 2, 1) is a palindromic composition; (1, 2, 2, 3, 1) is non-palindromic. We denote the palindromic compositions of *a* into *b* parts by $C_p(a,b)$ and the non-palindromic compositions of *a* into *b* parts by $C_{np}(a,b)$, such that $C_p(a,b) \, \cup \, C_{np}(a,b) = C(a,b)$ and $C_p(a,b) \, \cap \, C_{np}(a,b) = \emptyset$.

The numbers of palindromic and non-palindromic compositions, $|C_p(a,b)|$ and $|C_{np}(a,b)|$, appear in Table 1. Palindromic compositions are counted by counting the ways to place breakpoints on the “left” half of a list of 1’s; breakpoints are then palindromically placed on the “right” half. Distinct cases exist depending on the parity of *a* and *b*. For non-palindromic compositions, we obtain $|C_{np}(a,b)|$ from $|C(a,b)|-|C_p(a,b)|$.

### Unlabeled Trees with *n* Leaves

Our approach to enumerating unlabeled galled trees extends the Wedderburn-Etherington enumeration of unlabeled trees with no galls. For unlabeled trees with no galls, the root of a tree with $n \geqslant 2$ leaves possesses two immediate subtrees. Assume without loss of generality that the number of leaves in the “left” subtree is greater than or equal to the number of leaves in the “right” subtree. If $n \geqslant 3$ is odd, then $U_n$, the number of unlabeled trees with *n* leaves, is obtained by considering the $\frac{n-1}{2}$ possible numbers of leaves *k* for the right subtree, for each *k* pairing all $U_k$ unlabeled trees with *k* leaves for the right subtree with all $U_{n-k}$ unlabeled trees with $n-k$ leaves for the left subtree. If *n* is even, then the enumeration is similar for $k \ne \frac{n}{2}$; if $k = \frac{n}{2}$, however, then we have ${U_{n/2} \atopwithdelims ()2}$ ways choosing two distinct subtrees for the left and right subtrees and $U_{n/2}$ ways of choosing two copies of the same subtree.

The recursion for $U_n$ is [e.g. Harding (1971), Felsenstein (2004)]:

1 $$\begin{aligned} U_n = {\left\{ \begin{array}{ll} 1, &{} n=1, \\ \sum _{k=1}^{\frac{n-1}{2}}U_kU_{n-k}, &{} \text {odd } n\geqslant 3, \\ \bigg {(}\sum _{k=1}^{\frac{n}{2}-1}U_kU_{n-k}\bigg {)} +\frac{U_{\frac{n}{2}}(U_{\frac{n}{2}}+1)}{2}, &{} \text {even } n. \end{array}\right. } \end{aligned}$$

With $U_n=0$, the generating function ${\mathcal {U}}(t)=\sum _{n \geqslant 0} U_n t^n$ for the $U_n$ satisfies ( Comtet 1974, p. 55):

2 $$\begin{aligned} {\mathcal {U}}(t) = t+ {\frac{1}{2}}{\mathcal {U}}^2(t) + {\frac{1}{2}}{\mathcal {U}}(t^2). \end{aligned}$$

The number of trees with no galls has exponential growth with $d_0 \rho ^{-n} n^{-\frac{3}{2}}$, for constants $d_0 \approx 0.3188$ and $1/\rho \approx 2.4833$ ( Harding 1971; Landau 1977; Flajolet and Sedgewick 2009, p. 65).

### The Maximum Number of Galls for Galled Trees with *n* Leaves

For a fixed number of leaves *n*, the number of galls that a galled tree can possess is constrained as a function of *n*. Because a gall contains at least three descendant subtrees—those descended from the two hybridizing nodes and the hybrid node—a minimum of $n=3$ leaves is required before a tree can possess a gall. Each successive addition of a gall then replaces one subtree with a minimum of three subtrees—those descended from the two hybridizing nodes and the hybrid node of the new gall—so that each gall adds at least two leaves. It follows that a galled tree with *n* leaves can have at most $\lfloor \frac{n-1}{2} \rfloor$ galls (Mathur and Rosenberg 2023).

## Unlabeled Galled Trees with *n* Leaves

We are now ready to enumerate unlabeled galled trees. We denote by $A_n$ the number of unlabeled galled trees with *n* leaves. Trees with $n=1$ or $n=2$ leaves have no galls: $A_1=U_1=1$ and $A_2=U_2=1$. To recursively evaluate $A_n$ for $n \geqslant 3$ leaves, we sum counts from two cases: (1) the root is not the top node of a gall; (2) the root is the top node of a gall. We count galled trees in the former case in $B_n$, with $B_1=B_2=1$, and we count galled trees in the latter case in $D_n$, with $D_1=D_2=0$. The goal is to evaluate

3 $$\begin{aligned} A_n=B_n+D_n. \end{aligned}$$

### Root is not a Top Node of a Gall

**Fig. 2:**
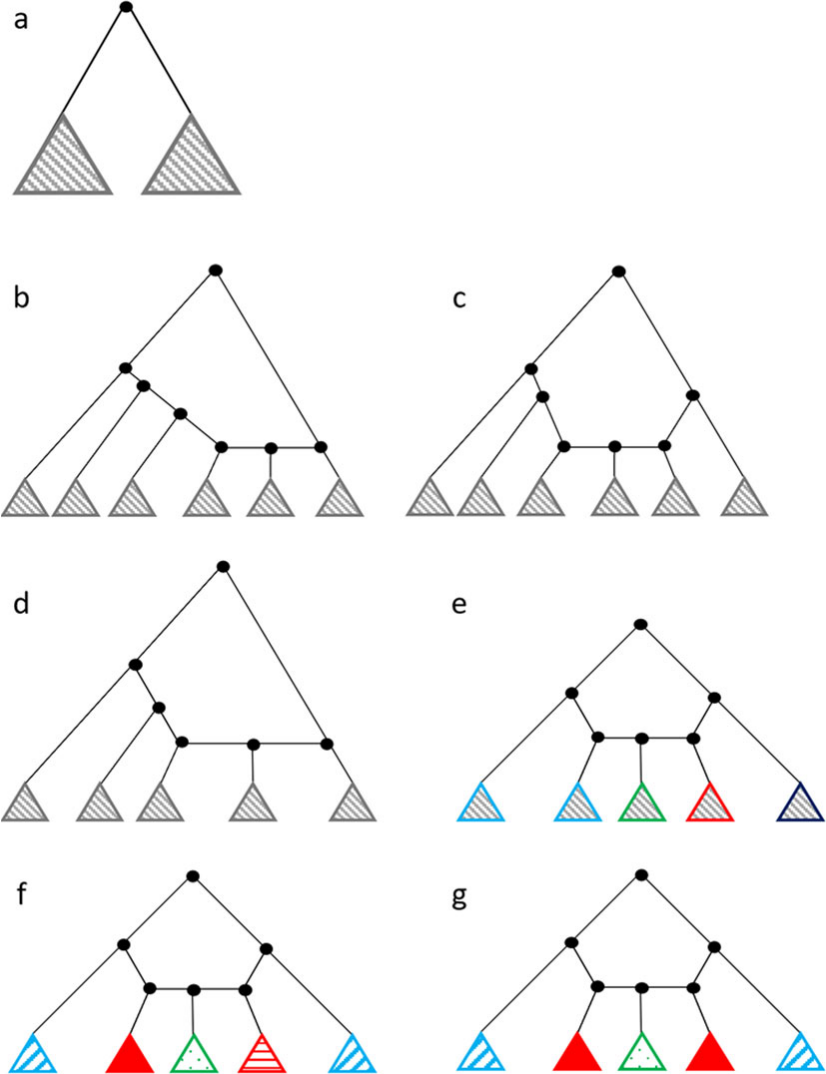
Recursive enumeration of rooted galled trees. Triangles indicate unspecified subtrees with at least one leaf. **A** The root is not a top node of a gall (Eq. 4). **B**, **C** The root is a top node of a gall with an even number of subtrees (Eq. 9). The two trees show the two cases with $k=6$: $(\ell , r)=(4,1)$ (**B**) and $(\ell ,r)=(3,2)$ (**C**). **D** The root is a top node of a gall with an odd number of subtrees and $r < \ell$ (Eq. 10). In this case, $k=5$ and $(\ell ,r)=(3,1)$. **E** The root is a top node of a gall with an odd number of subtrees, $r=\ell$, and the composition of *n* leaves descended from the root gall into $k=\ell +r+1$ parts representing *k* subtrees is non-palindromic (Eq. 11). In this case, $k=5$ and $(\ell ,r)=(2,2)$. Different outline colors for triangles indicate different numbers of leaves in associated subtrees. **F**, **G** The root is a top node of a gall with an odd number of subtrees, $r=\ell$, and the composition of *n* leaves into *k* parts is palindromic (Eq. 12). In both trees, $k=5$ and $(\ell ,r)=(2,2)$; trees with distinct (**F**) and identical (**G**) lists of galled subtrees for left and right subtrees are depicted. Different outline colors for triangles indicate different numbers of leaves in subtrees, and different patterns in the same color indicate different topologies with equally many leaves (Color figure online)

If the root is not the top node of a gall, then an unlabeled galled tree possesses two immediate subtrees of the root, each of which is itself an unlabeled galled tree (Fig. 2A). The number of unlabeled galled trees then follows a recursion analogous to Eq. 1:

4 $$\begin{aligned} B_n ={\left\{ \begin{array}{ll} 1, &{} n=1, \\ \sum _{m=1}^{\frac{n-1}{2}}A_mA_{n-m}, &{} \text {odd } n \geqslant 3, \\ \bigg {(}\sum _{m=1}^{\frac{n}{2}-1}A_mA_{n-m}\bigg {)} +\frac{A_{\frac{n}{2}}(A_{\frac{n}{2}}+1)}{2},&{} \text {even } n. \end{array}\right. } \end{aligned}$$

It is convenient to express $B_n$ in a form that considers compositions ${\textbf{c}}$ of *n* into 2 parts. For odd $n \geqslant 3$,

5 $$\begin{aligned} B_n=\frac{1}{2}\sum _{m=1}^{n-1}A_mA_{n-m}=\frac{1}{2}\sum _{{\textbf{c}} \in C(n,2)}A_{c_1}A_{c_2}. \end{aligned}$$

For even *n*,

6 $$\begin{aligned} B_n= \frac{1}{2}A_{\frac{n}{2}} + \frac{1}{2}\sum _{m=1}^{n-1}A_mA_{n-m}=\frac{1}{2}A_{\frac{n}{2}} + \frac{1}{2}\sum _{{\textbf{c}} \in C(n,2)}A_{c_1}A_{c_2}. \end{aligned}$$

### Root is a Top Node of a Gall

If the root is a top node of a gall, then the recursion is more complex. We first count subtrees of the root gall, equal to the count of all side nodes plus the hybrid node. Suppose the root gall contains *k* subtrees. We have the constraint $3 \leqslant k \leqslant n$, as a gall has at least 3 subtrees (of the left hybridizing node, hybrid node, and right hybridizing node), and the root gall can have as many as *n* subtrees, each ancestral to a single leaf.

Without loss of generality, we can assume that the number of right side nodes in the root gall, *r*, is less than or equal to the number of left side nodes $\ell$; owing to the existence of the hybrid node, $\ell + 1 + r = k$. We divide the case further based on the parity of *k*, writing

7 $$\begin{aligned} D_n = D_n^{(e)} + D_n^{(o)}, \end{aligned}$$

where $D_n^{(e)}$ and $D_n^{(o)}$ count unlabeled trees with *n* leaves in which the root is a top node and the number of descendant subtrees of the root gall is even and odd, respectively.

#### Even Number of Subtrees of the Root Gall

We consider each even value of *k*, $k=2a$ for $a=2,3,\ldots , \lfloor \frac{n}{2} \rfloor$. Given *k*, because $r \leqslant \ell$, *r* ranges from 1 to $\frac{k}{2}-1=a-1$. Because $\ell +1+r=k$ and *k* is even, we have the strict inequality $r < \ell$.

Consider the *k* subtrees in an order that proceeds from the most ancestral left side node descending through subsequent left side nodes to the left hybridizing node, then to the hybrid node, then the right hybridizing node, and then through ancestors to the most ancestral right side node (Fig. 2B, C). Once *k* and *r* have been specified, we consider all possible ways of placing the *n* leaves into the *k* subtrees of the gall: the compositions *C*(*n*, *k*) of *n* into *k* parts. For each composition ${\textbf{c}} = (c_1,c_2,\ldots ,c_k)$ in *C*(*n*, *k*), where $c_i$ is the value of the *i*th term, the number of galled trees is $\prod _{i=1}^k A_{c_i}$. We have

8 $$\begin{aligned} D_n^{(e)} = \sum _{a=2}^{\lfloor \frac{n}{2} \rfloor } \sum _{r=1}^{a-1} \sum _{{\textbf{c}} \in C(n,2a)} \prod _{i=1}^{2a} A_{c_i}. \end{aligned}$$

The summand $\sum _{{\textbf{c}} \in C(n,2a)} \prod _{i=1}^{2a} A_{c_i}$, representing the number of distinct lists of 2*a* subtrees with total number of leaves *n*, does not depend on *r*, the number of those subtrees descended from right side nodes. For $n \leqslant 2$, a sum from $a=2$ to $a=\lfloor \frac{n}{2} \rfloor$ is empty. We can therefore simplify Eq. 8 to obtain, for all $n \geqslant 1$,

9 $$\begin{aligned} D_n^{(e)} = \sum _{a=2}^{\lfloor \frac{n}{2} \rfloor } \bigg [ (a-1) \sum _{{\textbf{c}} \in C(n,2a)} \prod _{i=1}^{2a} A_{c_i} \bigg ]. \end{aligned}$$

#### Odd Number of Subtrees of the Root Gall

If *k* is odd, then we consider $k=2a+1$ for $a=1,2,\ldots , \lfloor \frac{n-1}{2} \rfloor$. In this case, with $r \leqslant \ell$, we have $1 \leqslant r \leqslant a$. With $\ell +1+r=k$ and *k* odd, $r=\ell$ is possible.

If $r<\ell$, then *r* ranges from 1 to $\frac{k-1}{2}-1=a-1$ (Fig. 2D). We follow the reasoning of the case of even *k* and find that this case contributes a number of galled trees equal to

10 $$\begin{aligned} \sum _{a=1}^{\lfloor \frac{n-1}{2} \rfloor } \bigg [ (a-1) \sum _{{\textbf{c}} \in C(n,2a+1)} \prod _{i=1}^{2a+1} A_{c_i} \bigg ]. \end{aligned}$$

Consider $r=\ell =a$. For non-palindromic compositions of *n* into *k* parts representing the *k* subtrees of the gall, an equivalent unlabeled galled tree is obtained by a mirror-image composition that corresponds to an exchange of left and right subtrees of the root gall (Fig. 2E). We therefore multiply by $\frac{1}{2}$ to account for the fact that each unlabeled galled tree is counted twice, so that the non-palindromic compositions of *n* contribute a number of galled trees equal to

11 $$\begin{aligned} \frac{1}{2} \sum _{a=1}^{\lfloor \frac{n-1}{2} \rfloor } \sum _{{\textbf{c}} \in C_{np}(n,2a+1)} \prod _{i=1}^{2a+1} A_{c_i}. \end{aligned}$$

With $r = \ell = a$, for a palindromic composition ${\textbf{c}}$ of *n* into *k* parts representing the *k* subtrees of the root gall (Fig. 2F, G), we can select two distinct lists of galled subtrees for the *a* left and the *a* right subtrees; the number of ways to do so is $(\prod _{i=1}^a A_{c_i})[(\prod _{i=1}^a A_{c_i}) - 1]/2$. Alternatively, we can select the same lists of galled subtrees for the *a* left and *a* right subtrees, in $\prod _{i=1}^a A_{c_i}$ ways. Any choices for the left and right subtrees can be combined with $A_{c_{a+1}}$ choices for the subtree of the hybrid node of the gall. The palindromic compositions produce a number of galled trees equal to

12 $$\begin{aligned} \sum _{a=1}^{\lfloor \frac{n-1}{2} \rfloor } \sum _{{\textbf{c}} \in C_{p}(n,2a+1)} \frac{\Big (\prod _{i=1}^{a} A_{c_i}\Big ) \Big [\big (\prod _{i=1}^{a} A_{c_i}\big ) + 1\Big ]A_{c_{a+1}}}{2}. \end{aligned}$$

Summing the three cases in Eqs. 10, 11, and 12, we obtain

13 $$\begin{aligned} D_n^{(o)}= & {} \sum _{a=1}^{\lfloor \frac{n-1}{2} \rfloor } \bigg [ \bigg ( \sum _{r=1}^{a-1} \sum _{{\textbf{c}} \in C(n,2a+1)} \prod _{i=1}^{2a+1} A_{c_i} \bigg ) \nonumber \\{} & {} + \bigg ( \frac{1}{2} \sum _{{\textbf{c}} \in C_{np}(n,2a+1)} \prod _{i=1}^{2a+1} A_{c_i} \bigg ) \nonumber \\{} & {} + \bigg ( \sum _{{\textbf{c}} \in C_{p}(n,2a+1)} \frac{\Big (\prod _{i=1}^{a} A_{c_i}\Big ) \Big [\big (\prod _{i=1}^{a} A_{c_i}\big ) + 1\Big ]A_{c_{a+1}}}{2} \bigg ) \bigg ]. \end{aligned}$$

We can simplify this expression further. For a palindromic composition ${\textbf{c}} \in C_p(n,2a+1)$, by definition of palindromic compositions, $c_i=c_{2a+2-i}$ for $i=1,2, \ldots , a$. $C(n,2a+1)$ is the disjoint union of $C_p(n,2a+1)$ and $C_{np}(n,2a+1)$. We can then write

$$\begin{aligned}{} & {} \bigg (\frac{1}{2}\sum _{{\textbf{c}} \in C_{np}(n,2a+1)} \prod _{i=1}^{2a+1} A_{c_i}\bigg ) + \bigg (\sum _{{\textbf{c}} \in C_{p}(n,2a+1)} \frac{\big (\prod _{i=1}^{a} A_{c_i})\big (\prod _{i=1}^{a} A_{c_i}\big )A_{c_{a+1}}}{2}\bigg )\\{} & {} \qquad = \frac{1}{2}\sum _{{\textbf{c}} \in C(n,2a+1)} \prod _{i=1}^{2a+1} A_{c_i}. \end{aligned}$$

Equation 13 then becomes

$$\begin{aligned} D_n^{(o)}= & {} \sum _{a=1}^{\lfloor \frac{n-1}{2} \rfloor }\bigg {[} \Big [\Big (\sum _{r=1}^{a-1}1 \Big )+\frac{1}{2}\Big ] \bigg (\sum _{{\textbf{c}} \in C(n,2a+1)} \prod _{i=1}^{2a+1} A_{c_i}\bigg ) +\bigg (\frac{1}{2}\sum _{{\textbf{c}} \in C_p(n,2a+1)} \prod _{i=1}^{a+1} A_{c_i}\bigg )\bigg {]}. \end{aligned}$$

Note that for $n \leqslant 2$, a sum from $a=1$ to $\lfloor \frac{n-1}{2} \rfloor$ is empty. Therefore, for all $n \geqslant 1$,

14 $$\begin{aligned} D_n^{(o)} = \sum _{a=1}^{\lfloor \frac{n-1}{2} \rfloor }\bigg {[} \bigg (a - \frac{1}{2}\bigg )\bigg ( \sum _{{\textbf{c}} \in C(n,2a+1)} \prod _{i=1}^{2a+1} A_{c_i}\bigg ) +\bigg (\frac{1}{2}\sum _{{\textbf{c}} \in C_p(n,2a+1)} \prod _{i=1}^{a+1} A_{c_i} \bigg )\bigg {]}.\qquad \end{aligned}$$

### Summary

To summarize the enumeration, the desired number of unlabeled galled trees with *n* leaves, $A_n$, can be calculated in Eq. 3 by summing Eqs. 9 and 14 in Eq. 7, and then adding the result to $B_n$ from Eq. 4.

We simplify by writing the sum of $D_n^{(e)}$ and the first term of $D_n^{(o)}$ in one expression. When adding the even-*k* terms in $D_n^{(e)}$ and the odd-*k* terms $D_n^{(o)}$, *k* now ranges from 3 to *n*, considering even values of *k* with $k=2a$ and $a=2,3,\ldots , \lfloor \frac{n}{2} \rfloor$, and odd values of *k* with $k=2a+1$ and $a=1,2,\ldots , \lfloor \frac{n-1}{2} \rfloor$. For $k=2a$, $a-1 = \frac{k-2}{2}$, and for $k=2a+1$, $a-\frac{1}{2} = \frac{k-2}{2}$ as well.

Recalling Eqs. 5 and 6, we can now write a simplified expression for the recursion for $A_n$. First, $A_1=1$. If the number of leaves *n* of an unlabeled galled tree is an odd value $n\geqslant 3$, then

15 $$\begin{aligned} A_n{} & {} = \frac{1}{2}\bigg {[}\bigg (\sum _{{\textbf{c}} \in C(n,2)}\prod _{i=1}^{2}A_{c_i}\bigg ) + \bigg (\sum _{k=3}^{n}(k-2)\sum _{{\textbf{c}} \in C(n,k)}\prod _{i=1}^{k}A_{c_i} \bigg )\nonumber \\{} & {} \quad \ + \bigg ( \sum _{a=1}^{\lfloor \frac{n-1}{2} \rfloor }\sum _{{\textbf{c}} \in C_p(n,2a+1)} \prod _{i=1}^{a+1} A_{c_i}\bigg ) \bigg {]}. \end{aligned}$$

If the number of leaves *n* is even, then an extra term appears:

16 $$\begin{aligned} A_n{} & {} = \frac{1}{2}\bigg {[}\bigg (\sum _{{\textbf{c}} \in C(n,2)}\prod _{i=1}^{2}A_{c_i}\bigg ) + A_{\frac{n}{2}} + \bigg ( \sum _{k=3}^{n}(k-2)\sum _{{\textbf{c}} \in C(n,k)}\prod _{i=1}^{k}A_{c_i}\bigg )\nonumber \\{} & {} \quad \ + \bigg ( \sum _{a=1}^{\lfloor \frac{n-1}{2} \rfloor }\sum _{{\textbf{c}} \in C_p(n,2a+1)}\prod _{i=1}^{a+1} A_{c_i}\bigg ) \bigg {]}. \end{aligned}$$

### Example

To illustrate the recursive enumeration of rooted galled trees, we enumerate the $A_5=20$ rooted galled trees with 5 leaves. The base case in Eqs. 15 and 16 is $A_1=1$. To evaluate $A_5$, we first evaluate $A_2$, $A_3$, and $A_4$.

#### Trees with Two Leaves

Only one galled tree has two leaves: the 2-leaf tree with no galls (Table 2). Equation 16 recovers this result: $B_2 = A_1(A_1+1)/2 = 1$ (Eq. 4), $D_2^{(e)}=0$ (Eq. 9), and $D_2^{(o)}=0$ (Eq. 14), so that $A_2=B_2 + D_2^{(e)} + D_2^{(o)} = 1$.

#### Trees with Three Leaves

For $n=3$, there are two galled trees (Table 2). Using Eqs. 4, 9, and 14, we have

$$\begin{aligned} B_3= & {} A_1 A_2 = 1 \\ D_4^{(e)}= & {} 0 \\ D_4^{(o)}= & {} \frac{1}{2}A_1 A_1 A_1 + \frac{1}{2}A_1 A_1 = 1. \end{aligned}$$

Summing $B_3=1$, representing the tree with $n=3$ leaves and no galls, $D_3^{(e)}=0$, and $D_3^{(o)}=1$ for the unique tree containing a gall, we have $A_3=2$.

#### Trees with Four Leaves

For $n=4$, the number of galled trees is 6. We use Eqs. 4, 9, and 14 to obtain

$$\begin{aligned} B_4= & {} A_1 A_3 + \frac{1}{2}A_2 (A_2 + 1) = 3 \\ D_4^{(e)}= & {} A_1 A_1 A_1 A_1 = 1 \\ D_4^{(o)}= & {} \frac{1}{2}(A_2 A_1 A_1 + A_1 A_2 A_1 + A_1 A_1 A_2) + \frac{1}{2} A_1 A_2 = 2. \end{aligned}$$

$B_4$ counts trees 1 to 3 in Table 2. $D_4^{(e)}$ counts the unique tree with $k=4$ subtrees of the root gall, tree 6. $D_4^{(o)}$ counts trees with $k=3$ subtrees of the root gall, trees 4 and 5. Summing the three quantities, $A_4=6$.

**Table 2:** Rooted unlabeled galled trees with at most 5 leaves

Number of leaves	Tree number	Number of galls	Galled tree
1	1	0	
2	1	0	
3	1	0	
3	2	1	
4	1	0	
4	2	1	
4	3	0	
4	4	1	
4	5	1	
4	6	1	
5	1	0	
5	2	1	
5	3	0	
5	4	1	
5	5	1	
5	6	1	
5	7	0	
5	8	1	
5	9	1	
5	10	2	
5	11	1	
5	12	1	
5	13	1	
5	14	2	
5	15	1	
5	16	1	
5	17	1	
5	18	1	
5	19	1	
5	20	1	

Galled trees with different numbers of galls appear in different colors (0, black; 1, orange; 2, purple). For each number of leaves *n*, we enumerate galled trees in a canonical order. We recursively proceed through trees in which the root is not a top node of a gall, incrementing the number of leaves in the right subtree. Next, for trees in which the root is a top node, we proceed in increasing order of the number of subtrees of the root gall; for fixed numbers of subtrees, we proceed in dictionary order of $(\ell , r)$ values; for fixed $(\ell , r)$, we use reverse dictionary order of the compositions of leaves into subtrees of the gall. The canonical order is used in proceeding through subtrees of a fixed size

#### Trees with Five Leaves

We are now ready for the calculation of $A_5$, which produces $A_5=20$ galled trees with five leaves (Table 2).

$$\begin{aligned} B_5= & {} A_1 A_4 + A_2 A_3 = 8 \\ D_5^{(e)}= & {} A_2 A_1 A_1 A_1 + A_1 A_2 A_1 A_1 + A_1 A_1 A_2 A_1 + A_1 A_1 A_1 A_2 = 4 \\ D_5^{(o)}= & {} \Big [\frac{1}{2}(A_3 A_1 A_1 + A_2 A_2 A_1 + A_2 A_1 A_2 + A_1 A_3 A_1 + A_1 A_2 A_2 + A_1 A_1 A_3)\\{} & {} \quad + \frac{1}{2} (A_2 A_1 + A_1 A_3) \Big ] + \Big (\frac{3}{2}A_1 A_1 A_1 A_1 A_1 + \frac{1}{2}A_1 A_1 A_1 \Big ) = 8. \end{aligned}$$

$B_5$ enumerates trees in which the root is not a top node of a gall, trees 1 to 8 for $n=5$ in Table 2. $D_5^{(e)}$ enumerates trees with $k=4$ subtrees of the root gall, trees 15 to 18. $D_5^{(o)}$ enumerates trees for which the root gall has $k=3$ (trees 9 to 14) or $k=5$ subtrees (trees 19 and 20). $B_5$, $D_5^{(e)}$, and $D_5^{(o)}$ sum to $A_5=20$.

## Unlabeled Galled Trees with *n* Leaves and *g* Galls

A salient feature of a galled tree is its number of galls. Having enumerated unlabeled galled trees with *n* leaves, we now proceed to subdivide the calculation according to the number of galls: the number of galled trees with *n* leaves is a sum over *g* from 0 to $\lfloor \frac{n-1}{2} \rfloor$ of the number of galled trees with *n* leaves and *g* galls.

We denote by $E_{n,g}$ the number of galled trees with *n* leaves and *g* galls. Because the maximum number of galls with *n* leaves is $\lfloor \frac{n-1}{2} \rfloor$ (Sect. 2.4), we define $E_{n,g}=0$ for $g > \lfloor \frac{n-1}{2} \rfloor$. As the unique galled tree with $n=1$ leaf has no galls, the base case is $E_{1,0}=1$.

Again we separate two cases: (1) the root is not the top node of a gall, and (2) the root is the top node of a gall. For the former case, we denote the count by $P_{n,g}$, with $P_{1,0}=P_{2,0}=1$. For the latter case, we denote the count by $R_{n,g}$, with $R_{1,g}=R_{2,g}=0$ for all *g*. We seek to obtain $E_{n,g}=P_{n,g}+R_{n,g}$. We use reasoning that parallels the case in which we do not keep track of the number of galls (Sect. 3).

Note that when summing over all possible values of *g*, for $n \geqslant 1$, we have

$$\begin{aligned} A_n= & {} \sum _{g=0}^{\lfloor \frac{n-1}{2} \rfloor } E_{n,g} \\ B_n= & {} \sum _{g=0}^{\lfloor \frac{n-1}{2} \rfloor } P_{n,g} \\ D_n= & {} \sum _{g=0}^{\lfloor \frac{n-1}{2} \rfloor } R_{n,g}. \end{aligned}$$

### Root is not a Top Node of a Gall

If the root is not a top node, then a tree with $n \geqslant 2$ leaves and *g* galls can be decomposed into two subtrees. We assign one of these trees *m* leaves, $1 \leqslant m \leqslant \lfloor \frac{n}{2} \rfloor$, and *h* galls, $0 \leqslant h \leqslant \min (g, \lfloor \frac{m-1}{2} \rfloor )$. If *n* and *g* are both even, then it is possible for the two subtrees to be identical. Similarly to Eq. 4, we have

17 $$\begin{aligned} P_{n,g} = {\left\{ \begin{array}{ll} 1, &{} (n,g)=(1,0), \\ \sum _{m=1}^{\frac{n-1}{2}} \sum _{h=0}^{\min (g, \lfloor \frac{m-1}{2} \rfloor )} E_{m,h} E_{n-m,g-h}, &{} \text {odd } n \geqslant 3, \\ \sum _{m=1}^{\frac{n}{2}} \sum _{h=0}^{\min (g, \lfloor \frac{m-1}{2} \rfloor )} E_{m,h} E_{n-m,g-h}, &{} \text {even } n \text { and odd } g\geqslant 1, \\ \bigg {(}\sum _{m=1}^{\frac{n}{2}-1} \sum _{h=0}^{\min (g, \lfloor \frac{m-1}{2} \rfloor )} E_{m,h} E_{n-m,g-h}\bigg {)}&{} \\ \quad +\frac{E_{\frac{n}{2},\frac{g}{2}}(E_{\frac{n}{2},\frac{g}{2}}+1)}{2},&{}\text {even } n \text { and even } g. \end{array}\right. }\nonumber \\ \end{aligned}$$

Note that in this equation, we can replace $\min (g, \lfloor \frac{m-1}{2} \rfloor )$ with *g*; for $\lfloor \frac{m-1}{2} \rfloor < h \leqslant g$, $E_{m,h}$ in the summand is zero, as a tree with *m* leaves has at most $\lfloor \frac{m-1}{2} \rfloor$ galls.

We write another expression for $P_{n,g}$ by considering compositions of *n* into two parts representing the numbers of leaves in the left and right subtrees. We also decompose *g*; because entries in a composition are strictly positive, we consider compositions of $g+2$, noting that each entry of the composition exceeds the associated number of galls by 1.

For (*n*, *g*) in which *n* or *g* is odd and $n \geqslant 2$, similarly to Eq. 5,

18 $$\begin{aligned} P_{n,g} = \frac{1}{2}\sum _{{\textbf{c}} \in C(n,2)}\sum _{{\textbf{d}} \in C(g+2,2)}E_{c_1,d_{1}-1}E_{c_2,d_{2}-1}. \end{aligned}$$

For (*n*, *g*) both even, as in Eq. 6,

19 $$\begin{aligned} P_{n,g} = \frac{1}{2}E_{\frac{n}{2},\frac{g}{2}} + \frac{1}{2}\sum _{{\textbf{c}} \in C(n,2)}\sum _{{\textbf{d}} \in C(g+2,2)}E_{c_1,d_{1}-1}E_{c_2,d_{2}-1}. \end{aligned}$$

### Root is a Top Node of a Gall

In the case of a root gall, we distribute among the subtrees of the root gall $g-1$ galls, as one of the *g* galls is the root gall. We again distinguish between even and odd numbers of subtrees of the root gall, writing $R_{n,g}=R_{n,g}^{(e)}+R_{n,g}^{(o)}$, where $R_{n,g}^{(e)}$ gives the number of trees with *n* nodes and *g* galls in which the root is a top node and the root gall has an even number of descendant subtrees, and $R_{n,g}^{(o)}$ gives the corresponding number of trees with an odd number of descendant subtrees of the root gall. We follow our reasoning of Sects. 3.2.1 and 3.2.2.

#### Even Number of Subtrees of the Root Gall

Suppose the number of the subtrees of the root gall is even, $k=2a$, $a=2,3,\ldots ,\lfloor \frac{n}{2} \rfloor$. As in Sect. 3.2.1, given *k*, the number of right side nodes of the root gall, *r*, ranges from 1 to $\frac{k}{2}-1=a-1$.

Here, however, we consider all ways of distributing $g-1$ galls across $k=2a$ subtrees. Just as *n* leaves are placed into 2*a* subtrees by a composition of *n* into 2*a* parts, $g-1$ galls are placed into 2*a* subtrees by a composition of $g-1+2a$ into 2*a* parts. By decomposing $g-1+2a$, we allow for the possibility of 0 galls in a subtree; in a composition ${\textbf{d}}$ of $g-1+2a$, the number of galls in entry $d_i$ is $d_i-1$.

For all (*n*, *g*) with $n \geqslant 1$ and $0 \leqslant g \leqslant \lfloor \frac{n-1}{2} \rfloor$, the resulting number of trees is similar to Eq. 9:

20 $$\begin{aligned} R_{n,g}^{(e)}=\sum _{a=2}^{\lfloor \frac{n}{2} \rfloor } \bigg [ (a-1) \sum _{{\textbf{c}} \in C(n,2a)} \sum _{{\textbf{d}}\in C(g-1+2a,2a)} \prod _{i=1}^{2a} E_{c_i,d_i-1} \bigg ]. \end{aligned}$$

#### Odd Number of Subtrees of the Root Gall

For an odd number of subtrees of the root gall $k=2a+1$, $a=1,2,\ldots ,\lfloor \frac{n-1}{2} \rfloor$, as in Sect. 3.2.2, *r* ranges from 1 to *a*. Again we consider $(\ell ,r)$ with $r<\ell$ (as in Eq. 10) and add the $r=\ell$ case (as in Eqs. 11 and 12).

If $r<\ell$, then similarly to the case of even *k*, the number of galled trees with *k* subtrees descended from a root gall of a tree with $n \geqslant 1$ leaves and $0 \leqslant g \leqslant \lfloor \frac{n-1}{2} \rfloor$ galls is

21 $$\begin{aligned} \sum _{a=1}^{\lfloor \frac{n-1}{2} \rfloor } \bigg [ (a-1) \sum _{{\textbf{c}} \in C(n,2a+1)} \sum _{{\textbf{d}}\in C(g-1+2a+1,2a+1)} \prod _{i=1}^{2a+1} E_{c_i,d_i-1} \bigg ]. \end{aligned}$$

If $r=\ell =a$, then we again distinguish between non-palindromic and palindromic compositions of *n* leaves into the *k* subtrees. Non-palindromic compositions do not result in symmetric trees, irrespective of the way the galls are placed across the subtrees. Therefore, considering only the non-palindromic compositions, similarly to Eq. 11, the number of galled trees with $n \geqslant 1$ leaves and $g \geqslant 0$ galls is

22 $$\begin{aligned} \frac{1}{2} \sum _{a=1}^{\lfloor \frac{n-1}{2} \rfloor } \sum _{{\textbf{c}} \in C_{np}(n,2a+1)} \sum _{{\textbf{d}}\in C(g-1+2a+1,2a+1)} \prod _{i=1}^{2a+1} E_{c_i,d_i-1}. \end{aligned}$$

Finally, for the palindromic compositions of *n* leaves with *k* odd and $r=\ell =a$, we distinguish between cases with palindromic and non-palindromic compositions describing the placement of the *g* galls across *k* subtrees. For the non-palindromic compositions, similarly to Eq. 22, the number of trees is

23 $$\begin{aligned} \frac{1}{2} \sum _{a=1}^{\lfloor \frac{n-1}{2} \rfloor } \sum _{{\textbf{c}} \in C_{p}(n,2a+1)} \sum _{{\textbf{d}}\in C_{np}(g-1+2a+1,2a+1)} \prod _{i=1}^{2a+1} E_{c_i,d_i-1}. \end{aligned}$$

If both the composition of *n* leaves and the composition of $g-1$ galls are palindromic, then, as in our reasoning for Eq. 12, we can choose either two distinct or two identical lists of subtrees for the *a* left subtrees and the *a* right subtrees, and the number of trees is

24 $$\begin{aligned}{} & {} \sum _{a=1}^{\lfloor \frac{n-1}{2} \rfloor } \sum _{{\textbf{c}} \in C_{p}(n,2a+1)}\sum _{{\textbf{d}} \in C_p(g-1+2a+1,2a+1)}\nonumber \\{} & {} \qquad \times \frac{\Big (\prod _{i=1}^{a} E_{c_i,d_i-1}\Big ) \Big [\big (\prod _{i=1}^{a} E_{c_i,d_i-1}\big ) + 1\Big ]E_{c_{a+1},d_{a+1}-1}}{2}. \end{aligned}$$

We sum Eqs. 21, 22, 23, and 24 to obtain

25 $$\begin{aligned} R_{n,g}^{(o)}= & {} \sum _{a=1}^{\lfloor \frac{n-1}{2} \rfloor } \bigg [\bigg (a-\frac{1}{2} \bigg ) \bigg ( \sum _{{\textbf{c}} \in C(n,2a+1)} \sum _{{\textbf{d}} \in C(g-1+2a+1,2a+1)} \prod _{i=1}^{2a+1} E_{c_i,d_i-1}\bigg ) \nonumber \\{} & {} + \bigg ( \frac{1}{2} \sum _{{\textbf{c}} \in C_p(n,2a+1)} \sum _{{\textbf{d}} \in C_p(g-1+2a+1,2a+1)} \prod _{i=1}^{a+1} E_{c_i,d_i-1} \bigg ) \bigg ]. \end{aligned}$$

### Summary

As $E_{n,g}=P_{n,g}+R_{n,g}^{(e)}+R_{n,g}^{(o)}$, we summarize by adding Eqs. 17, 20, and 25. $E_{1,0}=1$ and $E_{1,g}=0$ for $g \geqslant 1$. For (*n*, *g*) with $n \geqslant 2$ leaves and $0 \leqslant g \leqslant \lfloor \frac{n-1}{2} \rfloor$ galls, if *n* is odd, *g* is odd, or both *n* and *g* are odd, then

26 $$\begin{aligned} E_{n,g}= & {} \frac{1}{2}\bigg {[}\bigg {(}\sum _{{\textbf{c}} \in C(n,2)}\sum _{{\textbf{d}} \in C(g+2,2)}\prod _{i=1}^2E_{c_i,d_{i}-1}\bigg {)} \nonumber \\{} & {} + \bigg {(}\sum _{k=3}^{n}(k-2)\sum _{{\textbf{c}}\in C(n,k)}\sum _{{\textbf{d}}\in C(g-1+k,k)}\prod _{i=1}^kE_{c_i,d_{i}-1}\bigg {)} \nonumber \\{} & {} + \bigg {(}\sum _{a=1}^{\lfloor \frac{n-1}{2} \rfloor }\sum _{{\textbf{c}} \in C_p(n,2a+1)}\sum _{{\textbf{d}} \in C_p(g-1+2a+1,2a+1)}\prod _{i=1}^{a+1} E_{c_i,d_{i}-1}\bigg {)}\bigg {]}. \end{aligned}$$

If both *n* and *g* are even, then an extra term appears:

27 $$\begin{aligned} E_{n,g}= & {} \frac{1}{2}\bigg {[}\bigg {(}\sum _{{\textbf{c}} \in C(n,2)}\sum _{{\textbf{d}} \in C(g+2,2)}\prod _{i=1}^2E_{c_i,d_{i}-1}\bigg {)} + E_{\frac{n}{2},\frac{g}{2}} \nonumber \\{} & {} + \bigg {(}\sum _{k=3}^{n}(k-2)\sum _{{\textbf{c}}\in C(n,k)}\sum _{{\textbf{d}}\in C(g-1+k,k)}\prod _{i=1}^kE_{c_i,d_{i}-1}\bigg {)} \nonumber \\{} & {} + \bigg {(}\sum _{a=1}^{\lfloor \frac{n-1}{2} \rfloor }\sum _{{\textbf{c}} \in C_p(n,2a+1)}\sum _{{\textbf{d}} \in C_p(g-1+2a+1,2a+1)}\prod _{i=1}^{a+1} E_{c_i,d_{i}-1}\bigg {)}\bigg {]}. \end{aligned}$$

### Example: 1 Gall

After the galled trees with no galls (Sect. 2.3), the next simplest case for enumeration of galled trees is the galled trees with only one gall. For this case, if there is no root gall, then the one gall must be in exactly one of the two subtrees descended from the root. The other subtree is a tree with no galls. If a root gall is present, then there are no other galls, and all subtrees descended from the root gall are trees with no galls.

For $n=1$, $E_{1,1}=0$. For $n \geqslant 2$, using the odd case Eq. 26, the first term of Eq. 26 when $g=1$ is

$$\begin{aligned} \frac{1}{2}\sum _{{\textbf{c}} \in C(n,2)}\sum _{{\textbf{d}} \in C(3,2)}\prod _{i=1}^2E_{c_i,d_{i}-1}{} & {} = \frac{1}{2}\sum _{c_1=1}^{n-1}\sum _{d_1=0}^{1}E_{c_1,d_1}E_{n-c_1,1-d_1} =\sum _{m=1}^{n-1}E_{m,0}E_{n-m,1}\\{} & {} = \sum _{m=1}^{n-1}U_mE_{n-m,1}. \end{aligned}$$

The second term is

$$\begin{aligned} \frac{1}{2}\sum _{k=3}^{n}(k-2)\sum _{{\textbf{c}}\in C(n,k)}\sum _{{\textbf{d}}\in C(k,k)}\prod _{i=1}^kE_{c_i,d_{i}-1}{} & {} = \frac{1}{2}\sum _{k=3}^{n}(k-2)\sum _{{\textbf{c}}\in C(n,k)}\prod _{i=1}^kE_{c_i,0}\\{} & {} = \frac{1}{2}\sum _{k=3}^{n}(k-2)\sum _{{\textbf{c}}\in C(n,k)}\prod _{i=1}^kU_{c_i}. \end{aligned}$$

The third term is

$$\begin{aligned} \frac{1}{2}\sum _{a=1}^{\lfloor \frac{n-1}{2} \rfloor }\sum _{{\textbf{c}} \in C_p(n,2a+1)}\sum _{{\textbf{d}} \in C_p(2a+1,2a+1)}\prod _{i=1}^{a+1} E_{c_i,d_{i}-1}{} & {} = \frac{1}{2}\sum _{a=1}^{\lfloor \frac{n-1}{2} \rfloor }\sum _{{\textbf{c}} \in C_p(n,2a+1)}\prod _{i=1}^{a+1} E_{c_i,0}\\{} & {} = \frac{1}{2}\sum _{a=1}^{\lfloor \frac{n-1}{2} \rfloor }\sum _{{\textbf{c}} \in C_p(n,2a+1)}\prod _{i=1}^{a+1} U_{c_i}. \end{aligned}$$

Summing the three terms, the number of galled trees with $n\geqslant 2$ leaves and $g=1$ gall is:

28 $$\begin{aligned} E_{n,1}{} & {} = \bigg ( \sum _{m=1}^{n-1}U_mE_{n-m,1} \bigg )\nonumber \\{} & {} \quad +\frac{1}{2}\bigg {[} \bigg (\sum _{k=3}^{n}(k-2) \sum _{{\textbf{c}} \in C(n,k)}\prod _{i=1}^{k}U_{c_i}\bigg ) + \bigg (\sum _{a=1}^{\lfloor \frac{n-1}{2} \rfloor }\sum _{{\textbf{c}} \in C_p(n,2a+1)}\prod _{i=1}^{a+1}U_{c_i}\bigg ) \bigg {]}.\qquad \end{aligned}$$

## Generating Functions

We now derive and analyze generating functions for $A_n$, the number of galled trees with *n* leaves, and $E_{n,1}$, the number of galled trees with *n* leaves and 1 gall. We also show that the exponential growth of $A_n$ proceeds faster with *n* than the exponential growth of $U_n$, the number of trees without galls—but that $E_{n,1}$ and $U_n$ follow the same exponential growth.

To analyze the generating functions, we will need the values of $A_n$ for small *n* and $E_{n,g}$ for small *n* and *g*. Hence, we use our recursions to exhaustively calculate the number of galled trees with *n* leaves, $A_n$ (Eqs. 15 and 16), and the number of galled trees with *n* leaves and $0 \leqslant g \leqslant \lfloor \frac{n-1}{2} \rfloor$ galls, $E_{n,g}$ (Eqs. 26 and 27). Considering *n* from 1 to 18, the numerical values appear in Table 3.

### Generating Function for $A_n$

Define a generating function ${\mathcal {A}}(t)=\sum _{n\geqslant 0} A_n t^n$, We rewrite Eqs. 15 and 16 in a single equation. To do so, we note $A_1=1$ and define $A_0=0$ and $A_n=0$ for non-integer values of *n*. For $n \geqslant 2$, we then have

29 $$\begin{aligned} A_n{} & {} = \frac{1}{2}\bigg [\bigg ( \sum _{m=0}^{n}A_m A_{n-m} \bigg ) + A_{\frac{n}{2}} + \bigg ( \sum _{k=3}^{n}(k-2)\sum _{{\textbf{c}} \in C(n,k)}\prod _{i=1}^{k} A_{c_i} \bigg )\nonumber \\{} & {} \qquad \ + \bigg ( \sum _{a=1}^{\lfloor \frac{n-1}{2} \rfloor }\sum _{{\textbf{c}} \in C_p(n,2a+1)}\prod _{i=1}^{a+1} A_{c_i} \bigg ) \bigg ]. \end{aligned}$$

We write the terms of the generating function with three components:

30 $$\begin{aligned} {\mathcal {A}}(t) = \sum _{n \geqslant 0}A_nt^n= & {} \frac{1}{2}\bigg [\underbrace{2t + \sum _{n\geqslant 2}\bigg (\Big (\sum _{m=0}^{n} A_m A_{n-m} \Big ) + A_{\frac{n}{2}}\bigg )t^n}_{{\mathcal {A}}_{\text {i}}(t)} \nonumber \\{} & {} +\underbrace{\sum _{n\geqslant 2} \bigg (\sum _{k=3}^{n}(k-2)\sum _{{\textbf{c}} \in C(n,k)}\prod _{i=1}^{k}A_{c_i} \bigg )t^n}_{{\mathcal {A}}_{\text {ii}}(t)} \nonumber \\{} & {} +\underbrace{\sum _{n\geqslant 2} \bigg (\sum _{a=1}^{\lfloor \frac{n-1}{2} \rfloor }\sum _{{\textbf{c}} \in C_p(n,2a+1)}\prod _{i=1}^{a+1} A_{c_i} \bigg )t^n}_{{\mathcal {A}}_{\text {iii}}(t)}\bigg ]. \end{aligned}$$

The first term has the form of twice the generating function for the Wedderburn-Etherington numbers (Eq. 2):

31 $$\begin{aligned} {\mathcal {A}}_{\text {i}}(t) = 2t+{\mathcal {A}}^2(t)+{\mathcal {A}}(t^2). \end{aligned}$$

For the second term,

32 $$\begin{aligned} {\mathcal {A}}_{\text {ii}}(t)= & {} \sum _{k\geqslant 3} (k-2)\sum _{n\geqslant k}\sum _{{\textbf{c}} \in C(n,k)}\prod _{i=1}^{k}(A_{c_i}t^{c_i}) \nonumber \\= & {} \sum _{k\geqslant 3}(k-2)\sum _{i_1\geqslant 0}\sum _{i_2\geqslant 0}\ldots \sum _{i_k\geqslant 0}A_{i_1} A_{i_2} \cdots A_{i_k}t^{i_1+i_2+\cdots +i_k} \end{aligned}$$

33 $$\begin{aligned}= & {} \sum _{k\geqslant 3}(k-2){\mathcal {A}}^k(t)=\sum _{m\geqslant 4}(m-3){\mathcal {A}}^{m-1}(t)\nonumber \\= & {} \bigg [\sum _{m\geqslant 1}(m-3){\mathcal {A}}^{m-1}(t)\bigg ]-[-2+(-1){\mathcal {A}}(t)] \nonumber \\= & {} \frac{{\mathcal {A}}(t)}{[1-{\mathcal {A}}(t)]^2}-\frac{2}{1-{\mathcal {A}}(t)}+2+{\mathcal {A}}(t). \end{aligned}$$

The step in Eq. 32 makes use of $A_0=0$. Equation 33 is obtained if and only if the sum in the previous step converges; that is, if and only if $|{\mathcal {A}}(t) |<1$.

Finally, for the third term,

34 $$\begin{aligned} {\mathcal {A}}_{\text {iii}}(t)= & {} \sum _{n\geqslant 3} \bigg (\sum _{a=1}^{\lfloor \frac{n-1}{2} \rfloor }\sum _{{\textbf{c}} \in C_p(n,2a+1)}\prod _{i=1}^{a+1} A_{c_i} \bigg )t^n \nonumber \\= & {} \sum _{m\geqslant 1}\sum _{a\geqslant m}\sum _{n\geqslant 2a+1}\sum _{{\textbf{c}} \in C(a,m)} \bigg (\prod _{i=1}^{m}(A_{c_i}t^{2c_i}) \bigg ) A_{n-2a}t^{n-2a} \nonumber \\= & {} \sum _{m\geqslant 1}\sum _{i_1\geqslant 0} \sum _{i_2\geqslant 0}\ldots \sum _{i_m\geqslant 0}A_{i_1}A_{i_2} \cdots A_{i_m}t^{2i_1+ 2i_2 + \cdots +2i_m} \sum _{\ell \geqslant 0}A_{\ell }t^{\ell } \end{aligned}$$

35 $$\begin{aligned}= & {} \sum _{m\geqslant 1}{\mathcal {A}}(t)\,{\mathcal {A}}^m(t^2) = \frac{{\mathcal {A}}(t)}{1-{\mathcal {A}}(t^2)}-{\mathcal {A}}(t). \end{aligned}$$

Equation 34 holds because $A_0=0$, and the last equality holds if and only if $|{\mathcal {A}}(t^2) |<1$.

Labeling the radius of convergence of ${\mathcal {A}}(t)$ by $\alpha$ and inserting into Eq. 30 the quantities in Eqs. 31, 33 and 35, for $0< |t| < \alpha$,

36 $$\begin{aligned} {\mathcal {A}}(t)= & {} 1+t+\frac{1}{2}{\mathcal {A}}^2(t)+\frac{1}{2}{\mathcal {A}}(t^2) - \frac{1}{1-{\mathcal {A}}(t)}+\frac{{\mathcal {A}}(t)}{2[1-{\mathcal {A}}(t)]^2} +\frac{{\mathcal {A}}(t)}{2[1-{\mathcal {A}}(t^2)]}.\nonumber \\ \end{aligned}$$

### Growth of $A_n$

We now address the asymptotic growth of $A_n$. In particular, we show that the number of galled trees grows exponentially faster in the number of leaves *n* than the corresponding number of trees without galls.

First, note that the radius of convergence $\alpha$ is a positive constant less than 1. The convergence radius of generating function ${\mathcal {U}}(t)$ for the $U_n$ (Eq. 2) is a value $\rho \approx 0.4027$, and in particular, $0< \rho < 1$ (p. 262 Landau 1977). Because $A_n>U_n$ for all $n \geqslant 3$, ${\mathcal {A}}(t) > {\mathcal {U}}(t)$ for all $0<t< \rho$. Hence, we have $\alpha \leqslant \rho < 1$; in the Appendix, we show $\alpha > 0$.

Also note that because $\alpha < 1$, $t^2 < t$ for $0< t < \alpha$. Because $|{\mathcal {A}}(t)| < 1$ for $0< t < \alpha$ and ${\mathcal {A}}(t)$ increases monotonically for $0< t < \alpha$, $|{\mathcal {A}}(t^2)| < 1$ for $0< t < \alpha$.

To find the asymptotic growth from the generating function for galled trees, ${\mathcal {A}}(t)$, we use the *asymptotics of implicit tree-like classes* theorem (Meir and Moon 1989a, b; Flajolet and Sedgewick 2009, pp. 467–468). This theorem describes the asymptotic growth of the coefficients of a generating function that is described implicitly, such as in Eq. 36. We write ${\mathcal {A}}(t) = \phi \big (t,{\mathcal {A}}(t) \big )$, and we denote ${\mathcal {A}}(t) = w$.

To use the theorem, we must first show that the function ${\mathcal {A}}(t)$, defined by $\phi (t,w)=\sum _{n,k}s_{n,k}t^nw^k$, belongs to the *smooth implicit-function schema*. Indeed, the necessary conditions are satisfied:

1. $\phi$ is analytic in *t* and *w* around 0 from Eq. 36 and the positive convergence radius $\alpha >0$.
2. $$A_0=0$$.
3. $$A_n \geqslant 0$$ for $$n \geqslant 1$$.
4. $s_{0,1} \ne 1$, which is verified by noting that the $t^0 w^1$ term in the right-hand side of Eq. 36 is equal to $-1 + \frac{1}{2} + \frac{1}{2} \sum _{m\geqslant 0} {\mathcal {A}}^m(t^2) = \frac{1}{2}\sum _{m\geqslant 1} {\mathcal {A}}^m(t^2) \ne 1$.
5. $s_{0,0}=0$, which follows from $\phi (0,0)={\mathcal {A}}(0)=0$, and $s_{n,k}\geqslant 0$, which is verified from the series expansion of Eq. 36.
6. From Eq. 33, there exists a coefficient $s_{n,k}>0$ for $n\geqslant 0$ and $k \geqslant 2$: for example, $s_{0,2}= \frac{1}{2} - 1 + \frac{1}{2} 2 = \frac{1}{2}$.
7. The last condition, which we show below, is that there are solutions $\alpha$ and $w_0$ for the characteristic system:
  37 $$\begin{aligned} \phi (\alpha ,w_0)= & {} w_0 \end{aligned}$$
  38 $$\begin{aligned} \phi _w(\alpha ,w_0)= & {} 1. \end{aligned}$$

According to the theorem, functions belonging to the smooth implicit-function schema converge at the solution to the characteristic system, where they possess a square-root singularity. We conclude that ${\mathcal {A}}(t)$ converges at $\alpha$, with ${\mathcal {A}}(\alpha )=w_0$, and that $[t^n]{\mathcal {A}}(t) \sim [\delta /(2\sqrt{\pi })]\alpha ^{-n} n^{-\frac{3}{2}}$, where

39 $$\begin{aligned} \delta = \sqrt{\frac{2\alpha \phi _t(\alpha ,w_0)}{\phi _{ww}(\alpha ,w_0)}}. \end{aligned}$$

It remains to show condition (7). We can write $\phi (t,w)$ as:

40 $$\begin{aligned} \phi (t,w)= & {} g_1(t)+\frac{1}{2}w^2-\frac{1}{1-w}+\frac{w}{2(1-w)^2}+wg_2(t), \end{aligned}$$

where $g_1(t)=1+t+\frac{1}{2}{\mathcal {A}}(t^2)$ and $g_2(t)= 1 /[2(1-{\mathcal {A}}(t^2))]$. Taking the derivative with respect to *w*, we have

41 $$\begin{aligned}{} & {} \phi _w(t,w)\nonumber \\{} & {} \quad = \frac{[-1 + 2g_2(t)] + [5-6g_2(t)] w + [-6+6g_2(t)]w^2+ [6-2g_2(t)] w^3-2w^4}{2(1-w)^3}. \nonumber \\ \end{aligned}$$

We do not know the value of ${\mathcal {A}}(\alpha ^2)$ that appears in $g_2(\alpha )$. ${\mathcal {A}}(t)$ is monotonically increasing with $t>0$; because $\alpha ^2$ is less than the radius of convergence $\alpha$, ${\mathcal {A}}$ converges at $\alpha ^2$ and ${\mathcal {A}}(\alpha ^2)$ is a finite constant. As shown above, ${\mathcal {A}}(\alpha ^2) < 1$. To find $(\alpha , w_0)$ numerically, we first note that Eq. 41 depends on *t* only through ${\mathcal {A}}(t^2)$. Hence, we can traverse values of $y={\mathcal {A}}(t^2)$, numerically solving Eq. 38 for the single variable *w* in terms of *y*. Solutions for *w* must satisfy $w > y$, as $w = {\mathcal {A}}(t) > {\mathcal {A}}(t^2)$ by the monotonicity of ${\mathcal {A}}(t)$.

Next, we see that Eq. 40 contains variables *w*, *y*, and *t*; using the pairs (*w*, *y*) obtained in the previous step, we numerically solve Eq. 37 for *t* in terms of *w* and *y*. In the third step, for each triple (*t*, *w*, *y*), we insert the value of *t* into the generating function $\sum _{n=1}^{25} A_n t^{2n}$, where values $A_1, A_2, \ldots , A_{25}$ are taken from Table 3; we retain triples with small $|y - \sum _{n=1}^{25} A_n t^{2n}|$. Note that in this step, we could instead have retained triples with small $|w - \sum _{n=1}^{25} A_n t^{n}|$; faster convergence of $\sum _{n=1}^{25} A_n t^{2n}$ compared to $\sum _{n=1}^{25} A_n t^{n}$ with a fixed number of known values of $A_n$ suggests that a more accurate result is obtained by use of *y* rather than *w*.

Finally, the best-fit triple (*t*, *w*, *y*) gives the numerical solution for $\big (\alpha , w_0, {\mathcal {A}}(\alpha ^2)\big )$, or (0.2073, 0.3550, 0.0450). We hence have $\alpha \approx 0.2073$ for the radius of convergence, and ${\mathcal {A}}(\alpha ) \approx 0.3550$. The radius $\alpha$ is indeed lower than $\rho \approx 0.4027$. Taking additional digits, the exponential order of the sequence $A_n$ is approximately $0.2073397^{-1} \approx 4.8230$, greater than that of the sequence $U_n$ for trees without galls ($0.4026975^{-1} \approx 2.4833$).

To calculate the asymptotic approximation to $A_n$, we evaluate the constant $\delta$. We have:

$$\begin{aligned} \phi _{ww}(t,w)= & {} 1 + \frac{3w}{(1-w)^4} \\ \phi _t(t,w)= & {} 1+t{\mathcal {A}}'(t^2)+wt\frac{{\mathcal {A}}'(t^2)}{[1-{\mathcal {A}}(t^2)]^2}. \end{aligned}$$

We numerically evaluate the derivative ${\mathcal {A}}'(\alpha ^2)$ from the first 25 terms by ${\mathcal {A}}'(\alpha ^2) \approx [\sum _{n=1}^{25}A_n(\alpha ^2)^{n}-\sum _{n=1}^{25}A_n(\alpha ^2-0.001)^{n}]/0.001$. Inserting $\alpha \approx 0.2073397$ for *t* and ${\mathcal {A}}(\alpha ) \approx 0.3550$ for *w*, we have $\phi _{ww}(\alpha ,w_0) \approx 7.1533$, ${\mathcal {A}}'(\alpha ^2) \approx 1.0981$, $\phi _t(\alpha ,w_0) \approx 1.3163$, $\delta \approx \sqrt{2 \cdot 0.2073 \cdot 1.3163 / 7.1533 } \approx 0.2762$ by Eq. 39, and

42 $$\begin{aligned} A_n = [t^n]{\mathcal {A}}(t)\sim 0.0779 (4.8230^n) n^{-\frac{3}{2}}. \end{aligned}$$

### Generating Function for $E_{n,1}$

We next find the generating function of $E_{n,1}$, ${\mathcal {E}}(t)=\sum _{n\geqslant 0} e_nt^n$, writing $e_n=E_{n,1}$. We define $e_0=0$, and recall that $e_1=0$ and that Eq. 28 applies for $n\geqslant 2$. We then have for $n\geqslant 1$

43 $$\begin{aligned} e_n = \bigg ( \sum _{m=0}^{n}U_m e_{n-m} \bigg ) +\frac{1}{2}\bigg {[} \bigg (\sum _{k=3}^{n}(k-2) \sum _{{\textbf{c}} \in C(n,k)}\prod _{i=1}^{k} U_{c_i}\bigg ) + \bigg (\sum _{a=1}^{\lfloor \frac{n-1}{2} \rfloor }\sum _{{\textbf{c}} \in C_p(n,2a+1)}\prod _{i=1}^{a+1} U_{c_i}\bigg ) \bigg {]}.\nonumber \\ \end{aligned}$$

We can now write

44 $$\begin{aligned} {\mathcal {E}}(t) = \sum _{n \geqslant 0} e_n t^n= & {} \frac{1}{2}\bigg [\underbrace{2 \sum _{n\geqslant 3}\bigg (\sum _{m=0}^{n}U_me_{n-m} \bigg )t^n}_{{\mathcal {E}}_{\text {i}}(t)} \nonumber \\{} & {} +\underbrace{\sum _{n\geqslant 3} \bigg (\sum _{k=3}^{n}(k-2)\sum _{{\textbf{c}} \in C(n,k)}\prod _{i=1}^{k}U_{c_i}\bigg )t^n}_{{\mathcal {E}}_{\text {ii}}(t)} \nonumber \\{} & {} +\underbrace{\sum _{n\geqslant 3} \bigg (\sum _{a=1}^{\lfloor \frac{n-1}{2} \rfloor }\sum _{{\textbf{c}} \in C_p(n,2a+1)}\prod _{i=1}^{a+1} U_{c_i}\bigg )t^n}_{{\mathcal {E}}_{\text {iii}}(t)}\bigg ]. \end{aligned}$$

As in the derivation of ${\mathcal {A}}{(t)}$, we calculate the three parts separately.

First, because $e_m=0$ for $m=0,1,2$,

45 $$\begin{aligned} {\mathcal {E}}_{\text {i}}(t)= & {} 2\sum _{n\geqslant 0}\bigg (\sum _{m=0}^{n}U_me_{n-m} \bigg )t^n \nonumber \\= & {} 2\sum _{m\geqslant 0}\sum _{n\geqslant m}(U_mt^m)(e_{n-m}t^{n-m}) \nonumber \\= & {} 2 \sum _{m\geqslant 0}\sum _{\ell \geqslant 0}(U_mt^m)(e_{\ell }t^{\ell })=\, 2{\mathcal {U}}(t) \, {\mathcal {E}}(t). \end{aligned}$$

For the second term, the derivation is identical to that of Eq. 33:

46 $$\begin{aligned} {\mathcal {E}}_{\text {ii}}(t)= & {} \frac{{\mathcal {U}}(t)}{[1-{\mathcal {U}}(t)]^2} - \frac{2}{1-{\mathcal {U}}(t)}+2+{\mathcal {U}}(t). \end{aligned}$$

Analogously to Eq. 33, Eq. 46 relies on a summation that can be completed if and only if $|{\mathcal {U}}(t)|<1$, that is, for $|t |< \rho$ (Landau 1977, Eqs. 4 and 5).

Finally, for the third term, following the derivation of Eq. 35,

47 $$\begin{aligned} {\mathcal {E}}_{\text {iii}}(t)= & {} \frac{{\mathcal {U}}(t)}{1-{\mathcal {U}}(t^2)}-{\mathcal {U}}(t), \end{aligned}$$

where the last equality holds if and only if $|{\mathcal {U}}(t^2)|<1$. Because $\rho <1$, $|t|^2 < |t|$ for $0< |t| < \rho$, and by the monotonicity of ${\mathcal {U}}(t)$ for $0< t < \rho$, $|{\mathcal {U}}(t^2)|< | {\mathcal {U}}(t) | <1$ for $0< |t |< \rho$.

Summarizing Eqs. 44, 45, 46, and 47, for $0< t < \rho$,

$$\begin{aligned} {\mathcal {E}}(t) = 1+{\mathcal {U}}(t)\,{\mathcal {E}}(t) - \frac{1}{1-{\mathcal {U}}(t)}+\frac{{\mathcal {U}}(t)}{2[1-{\mathcal {U}}(t)]^2} +\frac{{\mathcal {U}}(t)}{2[1-{\mathcal {U}}(t^2)]}. \end{aligned}$$

Solving for ${\mathcal {E}}(t)$, we have

48 $$\begin{aligned} {\mathcal {E}}(t) = \frac{1}{1-{\mathcal {U}}(t)} - \frac{1}{[1-{\mathcal {U}}(t)]^2}+\frac{{\mathcal {U}}(t)}{2[1-{\mathcal {U}}(t)]^3} +\frac{{\mathcal {U}}(t)}{2[1-{\mathcal {U}}(t)][1-{\mathcal {U}}(t^2)]}.\nonumber \\ \end{aligned}$$

### Growth of $E_{n,1}$

We now show that the asymptotic growth of the number of galled trees with one gall follows the asymptotic exponential growth of the number of trees with no galls. We also find the asymptotic approximation of $E_{n,1}$.

First, ${\mathcal {E}}(t) > {\mathcal {U}}(t)$. From the form of Eq. 48, ${\mathcal {E}}(t)$ converges if and only if $|{\mathcal {U}}(t)|<1$. It is shown in Eqs. 4 and 5 of Landau (1977) that $0< {\mathcal {U}}(t)<1$ for $0< t < \rho$, with $\lim _{t \rightarrow \rho ^-} {\mathcal {U}}(t) = 1$. We conclude that ${\mathcal {E}}(t)$ has the same radius of convergence $\rho$ as ${\mathcal {U}}(t)$. To find the asymptotic behavior of ${\mathcal {E}}(t)$, we notice that

49 $$\begin{aligned} {\mathcal {E}}(t)= & {} \frac{{\mathcal {U}}(t) \, \big [2{\mathcal {U}}(t)-1 \big ]}{2\big [1-{\mathcal {U}}(t) \big ]^3}+\frac{{\mathcal {U}}(t)}{2 \big [1-{\mathcal {U}}(t) \big ] \big [1-{\mathcal {U}}(t^2) \big ]}. \end{aligned}$$

As $t\rightarrow \rho ^-$, with ${\mathcal {U}}(t) \sim 1-\gamma \sqrt{1-t/\rho }$ and $\gamma \approx 1.1300$ (Flajolet and Sedgewick 2009, pp. 476–477), $1 - {\mathcal {U}}(t) \rightarrow 0$. Hence, the first of the two terms in Eq. 49 is the leading term as $t \rightarrow \rho ^-$, producing

50 $$\begin{aligned} {\mathcal {E}}(t)\sim & {} \frac{1}{2\gamma ^3(1-t/\rho )^\frac{3}{2}}. \end{aligned}$$

At this point, we seek to use *transfer theorems* to transfer the asymptotic equivalence for ${\mathcal {E}}(t)$ to an asymptotic equivalence for its coefficients. To do so, we note that ${\mathcal {U}}(t)$ satisfies the technical criterion that it is $\Delta$-analytic at $\rho$—that is, it is analytic in a domain $\Delta$ of particular shape around the singularity at $\rho$. The computation of ${\mathcal {E}}(t)$ from ${\mathcal {U}}(t)$ maintains the property that ${\mathcal {E}}(t)$ is $\Delta$-analytic with a singularity at $\rho$.

We can therefore use a transfer formula [Corollary VI.1, page 392 and Theorem VI.4, page 393 in Flajolet and Sedgewick (2009)], according to which, if *f*(*t*) is $\Delta$-analytic with a singularity at *b*, and $f(t)\sim (1-\frac{t}{b})^{-a}$ as $\frac{t}{b}\rightarrow 1$ with *t* in $\Delta$, and $a\notin \{0,-1,-2,\dots \}$, then the coefficients of *f* satisfy $[t^n]f(t)\sim n^{a-1} b^{-n} / \Gamma (a)$. Using Eq. 50, we apply the transfer formula to ${\mathcal {E}}(t)$ with $\rho$ in the role of *b* and $\frac{3}{2}$ for *a*, noting $\Gamma (\frac{3}{2}) = \sqrt{\pi }/{2}$:

51 $$\begin{aligned} E_{n,1}\sim & {} \frac{1}{2\gamma ^3\Gamma (\frac{3}{2})}n^{\frac{1}{2}}\rho ^{-n} = \frac{1}{\gamma ^3\sqrt{\pi }}n^{\frac{1}{2}}\rho ^{-n}. \end{aligned}$$

$E_{n,1}$ and $U_n$ have the same exponential growth. Whereas $U_n$ has subexponential term $0.3188 n^{-\frac{3}{2}}$, however, $E_{n,1}$ has larger subexponential term $0.3910 n^{\frac{1}{2}}$.

### Bivariate Generating Function for $E_{n,g}$

We now find the bivariate generating function ${\mathcal {A}}(t,u)=\sum _{n\geqslant 0}\sum _{g\geqslant 0}E_{n,g}t^nu^g$. First, note that $E_{0,g}=0$ for each $g \geqslant 0$. For $n=1$, $E_{1,0}=1$ and $E_{1,g}=0$ for $g \geqslant 1$. From the recursion for $E_{n,g}$ (Eqs. 26, 27), we get

52 $$\begin{aligned} {\mathcal {A}}(t,u)= & {} \frac{1}{2}\bigg {[}\underbrace{ 2t + \sum _{n\geqslant 2}\sum _{g\geqslant 0}\bigg ( \bigg ( \sum _{{\textbf{c}} \in C(n,2)}\sum _{{\textbf{d}} \in C(g+2,2)}\prod _{i=1}^2E_{c_i,d_{i}-1} \bigg ) + E_{\frac{n}{2},\frac{g}{2}}\bigg {)}t^nu^g}_{{\mathcal {A}}_{\text {i}}(t,u)} \nonumber \\{} & {} + \underbrace{\sum _{n\geqslant 2}\sum _{g\geqslant 0}\bigg {(}\sum _{k=3}^{n}(k-2)\sum _{{\textbf{c}}\in C(n,k)}\sum _{{\textbf{d}}\in C(g-1+k,k)}\prod _{i=1}^kE_{c_i,d_{i}-1}\bigg {)}t^nu^g}_{{\mathcal {A}}_{\text {ii}}(t,u)} \nonumber \\{} & {} + \underbrace{\sum _{n\geqslant 2}\sum _{g\geqslant 0}\bigg {(}\sum _{a=1}^{\lfloor \frac{n-1}{2} \rfloor }\sum _{{\textbf{c}} \in C_p(n,2a+1)}\sum _{{\textbf{d}} \in C_p(g-1+2a+1,2a+1)}\prod _{i=1}^{a+1} E_{c_i,d_{i}-1}\bigg {)}t^nu^g}_{{\mathcal {A}}_{\text {iii}}(t,u)}\bigg {]}, \nonumber \\ \end{aligned}$$

where $E_{m,\ell }=0$ if at least one of $(m,\ell )$ is not in ${\mathbb {N}}$.

We can solve to find an expression for ${\mathcal {A}}(t,u)$ in a manner similar to the solution for ${\mathcal {A}}(t)$. For the second and third terms, we have $\sum _{i=1}^k(d_i-1)=g-1$ and $\sum _{i=1}^{2a+1}(d_i-1)=g-1$; in these terms, the *g*th gall is the root gall. Therefore,

53 $$\begin{aligned} {\mathcal {A}}_i(t,u)= & {} 2t+ \sum _{n\geqslant 2}\sum _{g\geqslant 0}\sum _{m=0}^n\sum _{\ell =0}^gE_{m,\ell } E_{n-m,g-\ell }t^nu^g+\sum _{n\geqslant 0}\sum _{g\geqslant 0}E_{n,g}t^{2n}u^{2g} \nonumber \\= & {} 2t + \sum _{m\geqslant 0}\sum _{\ell \geqslant 0}E_{m,\ell }t^m u^{\ell }\sum _{n\geqslant m}\sum _{g\geqslant \ell }E_{n-m,g-\ell }t^{n-m}u^{g-\ell }+{\mathcal {A}}(t^2,u^2) \nonumber \\= & {} 2t + {\mathcal {A}}^2(t,u) + {\mathcal {A}}(t^2,u^2), \end{aligned}$$

54 $$\begin{aligned} {\mathcal {A}}_{ii}(t,u)= & {} \sum _{k \geqslant 3}(k-2)\sum _{n\geqslant k} \sum _{g \geqslant 1}\sum _{{\textbf{c}}\in C(n,k)}\sum _{{\textbf{d}}\in C(g-1+k,k)} \bigg ( \prod _{i=1}^k E_{c_i,d_i-1}t^{c_i}u^{d_i-1} \bigg ) u \nonumber \\= & {} u\sum _{k\geqslant 3}(k-2)\sum _{i_1\geqslant 0}\sum _{j_1\geqslant 0}E_{i_1,j_1}t^{i_1}u^{j_1} \sum _{i_2\geqslant 0}\sum _{j_2\geqslant 0}E_{i_2,j_2}t^{i_2}u^{j_2} \dots \sum _{i_k\geqslant 0}\sum _{j_k\geqslant 0}E_{i_k,j_k}t^{i_k}u^{j_k} \nonumber \\= & {} u\sum _{k\geqslant 3}(k-2){\mathcal {A}}^k(t,u) \nonumber \\= & {} u\bigg {[}\frac{{\mathcal {A}}(t,u)}{[1-{\mathcal {A}}(t,u)]^2} -\frac{2}{1-{\mathcal {A}}(t,u)}+2+{\mathcal {A}}(t,u)\bigg {]}, \end{aligned}$$

55 $$\begin{aligned} {\mathcal {A}}_{iii}(t,u)= & {} \sum _{m\geqslant 1}\sum _{a \geqslant m}\sum _{n\geqslant 2a+1}\sum _{b\geqslant 0}\sum _{g-1\geqslant 2b}\sum _{{\textbf{c}}\in C(a,m)}\nonumber \\{} & {} \quad \sum _{{\textbf{d}}\in C(b+m,m)} \bigg ( \prod _{i=1}^m E_{c_i,d_i-1}t^{2c_i}u^{2(d_i-1)} \bigg ) E_{n-2a,(g-1)-2b}t^{n-2a}u^{(g-1)-2b}u \nonumber \\= & {} u\sum _{m\geqslant 1}\sum _{i_1\geqslant 0}\sum _{j_1\geqslant 0}E_{i_1,j_1}t^{2i_1} u^{2j_1}\sum _{i_2\geqslant 0}\sum _{j_2\geqslant 0}E_{i_2,j_2}t^{2i_2}u^{2j_2}\dots \nonumber \\{} & {} \quad \sum _{i_m\geqslant 0}\sum _{j_m\geqslant 0}E_{i_m,j_m}t^{2i_m}u^{2j_m}\sum _{\ell \geqslant 0}\sum _{p \geqslant 0}E_{\ell ,p}t^{\ell }u^p \nonumber \\= & {} u\sum _{m\geqslant 1}{\mathcal {A}}(t,u){\mathcal {A}}^m(t^2,u^2) = u\bigg {[}\frac{{\mathcal {A}}(t,u)}{1-{\mathcal {A}}(t^2,u^2)}-{\mathcal {A}}(t,u)\bigg {]}. \end{aligned}$$

In summary, inserting Eqs. 53, 54, and 55 into Eq. 52,

56 $$\begin{aligned} {\mathcal {A}}(t,u)= & {} u+ t+\frac{1}{2}{\mathcal {A}}^2(t,u)+\frac{1}{2}{\mathcal {A}}(t^2,u^2) - \frac{u}{1-{\mathcal {A}}(t,u)}+\frac{u{\mathcal {A}}(t,u)}{2[1-{\mathcal {A}}(t,u)]^2} \nonumber \\{} & {} \quad +\frac{u{\mathcal {A}}(t,u)}{2[1-{\mathcal {A}}(t^2,u^2)]}. \end{aligned}$$

### The Distribution of the Number of Galled Trees with a Fixed Number of Leaves

The bivariate generating function ${\mathcal {A}}(t,u)$ provides a basis for studying the distribution of the number of galls across galled trees with *n* leaves. The approach follows a theorem concerning asymptotic distributions in Theorem 2.23 of Drmota (2009) and Proposition IX.17 on p. 682 of (Flajolet and Sedgewick 2009). We use the form of the theorem quoted in Theorem 2 of Bouvel et al. (2020), who considered labeled galled trees. We conclude that for a fixed number of leaves, the number of galled trees as a function of the number of galls *g* is asymptotically normally distributed with mean and variance linear in *n*.

Following Bouvel et al. (2020), we consider a power series *C*(*z*, *x*) in two variables that is defined implicitly as the solution of $C(z,x)=F\big (z,x,C(z,x)\big )$, where *F* satisfies certain conditions. We suppose $\{X_n\}$ is a sequence of random variables such that ${\mathbb {E}}[x^{X_n}]={[z^n]C(z,x)}/{[z^n]C(z,1)}$. Then $X_n$ is asymptotically normally distributed with a mean and variance that are linear multiples of *n* calculated from *F*.

In our scenario, *t*, *u*, and ${\mathcal {A}}$ play the roles of *z*, *x*, and *C*. ${\mathcal {A}}(t,u)$ is implicitly defined as a function of *t*, *u*, and ${\mathcal {A}}$ itself. With ${\mathcal {A}}(t,u) = \sum _{n\geqslant 0}\sum _{g\geqslant 0}E_{n,g} t^n u^g$, $X_n$ gives the random number of galls in a randomly selected galled tree with *n* leaves. Fixing the number of leaves *n* in ${\mathcal {A}}(t,u)$, this random variable satisfies

57 $$\begin{aligned} {\mathbb {E}}[u^{X_n}] = \frac{\sum _{g \geqslant 0} E_{n,g} t^n u^g}{\sum _{g \geqslant 0} E_{n,g} t^n} = \frac{\sum _{g \geqslant 0} E_{n,g} u^g}{\sum _{g \geqslant 0} E_{n,g} } = \frac{[t^n] {\mathcal {A}}(t,u)}{[t^n] {\mathcal {A}}(t,1)}. \end{aligned}$$

To conclude that random variable $X_n$—the random number of galls in a tree with *n* leaves—is normally distributed, it remains only to verify the conditions of the theorem.

Translating from the notation of Bouvel et al. (2020) and writing ${\mathcal {A}}(t,u)=\psi \big (t,u,{\mathcal {A}}(t,u) \big )$ with ${\mathcal {A}}(t,u)=w$ so that $w=\psi (t,u,w)$, we must show all of the following:

1. $\psi (t,u,w)$ is analytic in *t*, *u*, *w* around 0.
2. $$\psi (0,u,w) = 0$$.
3. $$\psi (t,u,0) \ne 0$$ for $$t>0$$.
4. All coefficients $[t^n u^g] \psi (t,u,w)$ are real and nonnegative.
5. Nonnegative solutions $(t,w)=(t_0,w_0)$ exist for the following pair of equations:
  $$\begin{aligned}{} & {} \text {i. } \psi (t,1,w) = w, \\{} & {} \text {ii. } \psi _{w}(t,1,w) = 1. \end{aligned}$$
6. The solutions satisfy:
  $$\begin{aligned}{} & {} \text {i. } \psi _{ww}(t_0,1,w_0) \ne 0, \\{} & {} \text {ii. } \psi _t(t_0,1,w_0) \ne 0. \end{aligned}$$

Condition 1 holds because $\psi$ is a quotient of analytic functions in *t*, *u*, *w* with denominator greater than 0 near (0, 0, 0). Condition 2 is met because ${\mathcal {A}}(t,u)=\sum _{n\geqslant 0}\sum _{g\geqslant 0}E_{n,g}t^nu^g =\sum _{n\geqslant 1}\sum _{g\geqslant 0}E_{n,g}t^nu^g$ (because $E_{0,g}=0$ for all $g\geqslant 0$) and so ${\mathcal {A}}(0,u) = \sum _{n\geqslant 1}\sum _{g\geqslant 0}0^nu^g=0$. Therefore,

$$\begin{aligned} \psi (0,u,{\mathcal {A}}){} & {} = u+0+\frac{1}{2}{\mathcal {A}}^2(0,u)+\frac{1}{2}{\mathcal {A}}(0^2,u^2) - \frac{u}{1-{\mathcal {A}}(0,u)}+\frac{u{\mathcal {A}}(0,u)}{2[1-{\mathcal {A}}(0,u)]^2}\nonumber \\{} & {} \quad +\frac{u{\mathcal {A}}(0,u)}{2[1-{\mathcal {A}}(0^2,u^2)]} \nonumber \\{} & {} = u+0+0+0-u+0+0=0. \end{aligned}$$

For condition 3, $\psi (t,u,0) = t + 0 + {\mathcal {A}}(t^2,u^2) - 0 + 0 + 0$, which is not equal to 0 for $t>0$. Condition 4 holds trivially from the definition of ${\mathcal {A}}(t,u)$. For conditions 5 and 6, we first show $\psi (t,1,w) = \phi (t,w)$. First,

$$\begin{aligned} \psi (t,1,{\mathcal {A}}(t,1)){} & {} = 1+t+\frac{1}{2}{\mathcal {A}}^2(t,1)+\frac{1}{2}{\mathcal {A}}(t^2,1^2) - \frac{1}{1-{\mathcal {A}}(t,1)}+\frac{1\cdot {\mathcal {A}}(t,1)}{2[1 -{\mathcal {A}}(t,1)]^2}\\{} & {} \quad \ +\frac{1\cdot {\mathcal {A}}(t,1)}{2[1-{\mathcal {A}}(t^2,1^2)]}. \end{aligned}$$

Next, we have

$$\begin{aligned} {\mathcal {A}}(t,1)= & {} \sum _{n \geqslant 0}\sum _{g\geqslant 0}E_{n,g}t^n1^g = \sum _{n \geqslant 0}\bigg (\sum _{g\geqslant 0}E_{n,g} \bigg ) t^n = \sum _{n \geqslant 0}A_nt^n = {\mathcal {A}}(t). \end{aligned}$$

We then have $\psi (t,1,w)=\phi (t,w)$. We have already shown conditions 5 and 6 in our analysis of function $\phi$.

With all the conditions demonstrated, we conclude that the random number of galls in a tree with *n* leaves is normally distributed.

## Numerical Results

The numerical results for the number of galled trees with *n* leaves and the number of galled trees with $0 \leqslant g \leqslant \lfloor \frac{n-1}{2} \rfloor$ galls suggest a number of simple observations (Table 3). First, for $g=0$, we recover the Wedderburn-Etherington numbers obtained from Eq. 1. For $n=1$ to 6, we obtain the values of $A_n$ and $E_{n,g}$ computed by Mathur and Rosenberg (2023). Finally, as *g* is bounded above by $g_{\max } = \lfloor \frac{n-1}{2} \rfloor$, pairs of consecutive values of *n*, an odd then an even integer, have the same number of values of *g* for which the number of galled trees $E_{n,g}$ is nonzero, namely $\lfloor \frac{n+1}{2} \rfloor$.

**Table 3:** Numbers of galled trees with specified numbers of leaves and galls

Number of	Total number	Number of trees with a fixed number of galls ($E_{n,g}$)								
leaves (*n*)	of trees ($A_n$)	$g=0$	$g=1$	$g=2$	$g=3$	$g=4$	$g=5$	$g=6$	$g=7$	$g=8$
1	1	1	–	–	–	–	–	–	–	–
2	1	1	–	–	–	–	–	–	–	–
3	2	1	1	–	–	–	–	–	–	–
4	6	2	4	–	–	–	–	–	–	–
5	20	3	15	2	–	–	–	–	–	–
6	72	6	48	18	–	–	–	–	–	–
7	272	11	148	107	6	–	–	–	–	–
8	1064	23	435	528	78	–	–	–	–	–
9	4271	46	1250	2295	661	19	–	–	–	–
10	17,497	98	3512	9185	4356	346	–	–	–	–
11	72,843	207	9726	34,503	24,564	3776	67	–	–	–
12	307,307	451	26,587	123,612	123,825	31,289	1543	–	–	–
13	1,310,792	983	71,975	426,218	574,149	216,501	20,720	246	–	–
14	5,643,555	2179	193,200	1,425,011	2,493,129	1,316,450	206,644	6942	–	–
15	24,493,270	4850	515,051	4,643,119	10,269,351	7,254,224	1,695,084	110,647	944	–
16	107,043,258	10,905	1,364,896	14,804,696	40,496,606	36,980,263	12,063,205	1,291,278	31,409	–
17	470,668,034	24,631	3,598,794	46,336,619	153,960,249	176,934,884	76,980,753	12,248,152	580,235	3717
18	2,080,681,402	56,011	9,447,028	142,720,317	567,348,929	803,058,979	450,309,678	99,840,890	7,756,699	142,871

Entries $E_{n,g}$ are computed recursively from Eqs. 26 and 27, and for fixed *n*, the entries in a row sum to the value of $A_n$ computed recursively from Eqs. 15 and 16. Additional values of $A_n$ used for approximating the generating function ${\mathcal {A}}(t)$ are $A_{19}=9,242,180,923$, $A_{20}=41,229,189,089$, $A_{21}=184,634,145,428$, $A_{22}=829,732,117,279$, $A_{23}=3,740,636,883,361$, $A_{24}=16,912,812,764,736$, and $A_{25}=76,673,344,515,050$

**Fig. 3:**
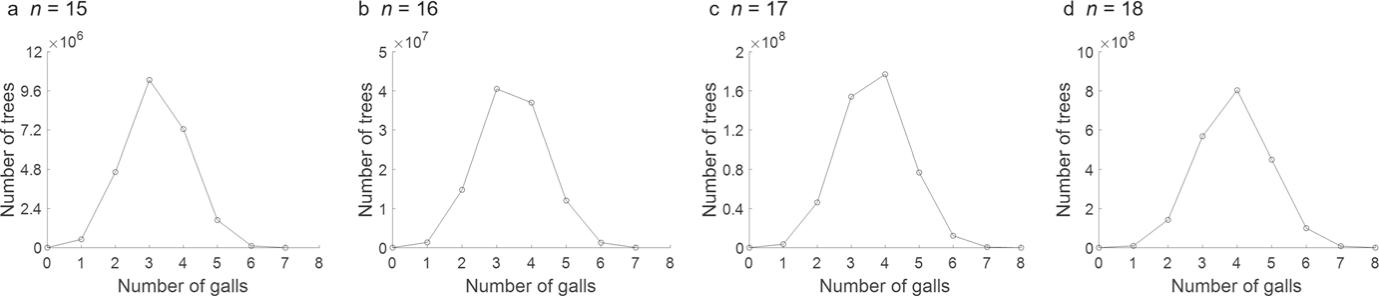
Number of galled trees as a function of the number of galls *g*, for fixed numbers of leaves *n*. **A** $n=15$. **B** $n=16$. **C** $n=17$. **D** $n=18$. Values are computed from Eqs. 26 and 27

Considering a fixed number of leaves $n \leqslant 18$, we comment informally on the number of galled trees across different values of *g*. The number of trees with at least one gall is larger than the number without galls. As *g* increases for fixed *n*, the number of trees increases to a maximum, then declines. For values of *n* for which the maximal number of galls is even ($n=1,2,5,6,9,10,13,14,17,18$), the largest number of trees occurs when the number of galls is $g_{\max }/2$, half of this maximum. When $g_{\max }$ is odd ($n=3,4,7,8,11,12,15,16$), the largest number of trees occurs at $g=(g_{\max }-1)/2$ or $g=(g_{\max }+1)/2$.

Figure 3 plots the number of trees for fixed *n* as a function of *g*, considering four consecutive values of *n* that represent the four cases possible for the parity of *n* and $g_{\max }$. The plots are somewhat symmetric; for $n=16$ and 17, a neighboring value of *g* produces a number of trees close to the maximum, and for $n=15$ and 18, the peak stands out more clearly. The patterns accord with the asymptotic normal distribution demonstrated for the number of galls as *n* increases (Sect. 5.6).

**Fig. 4:**
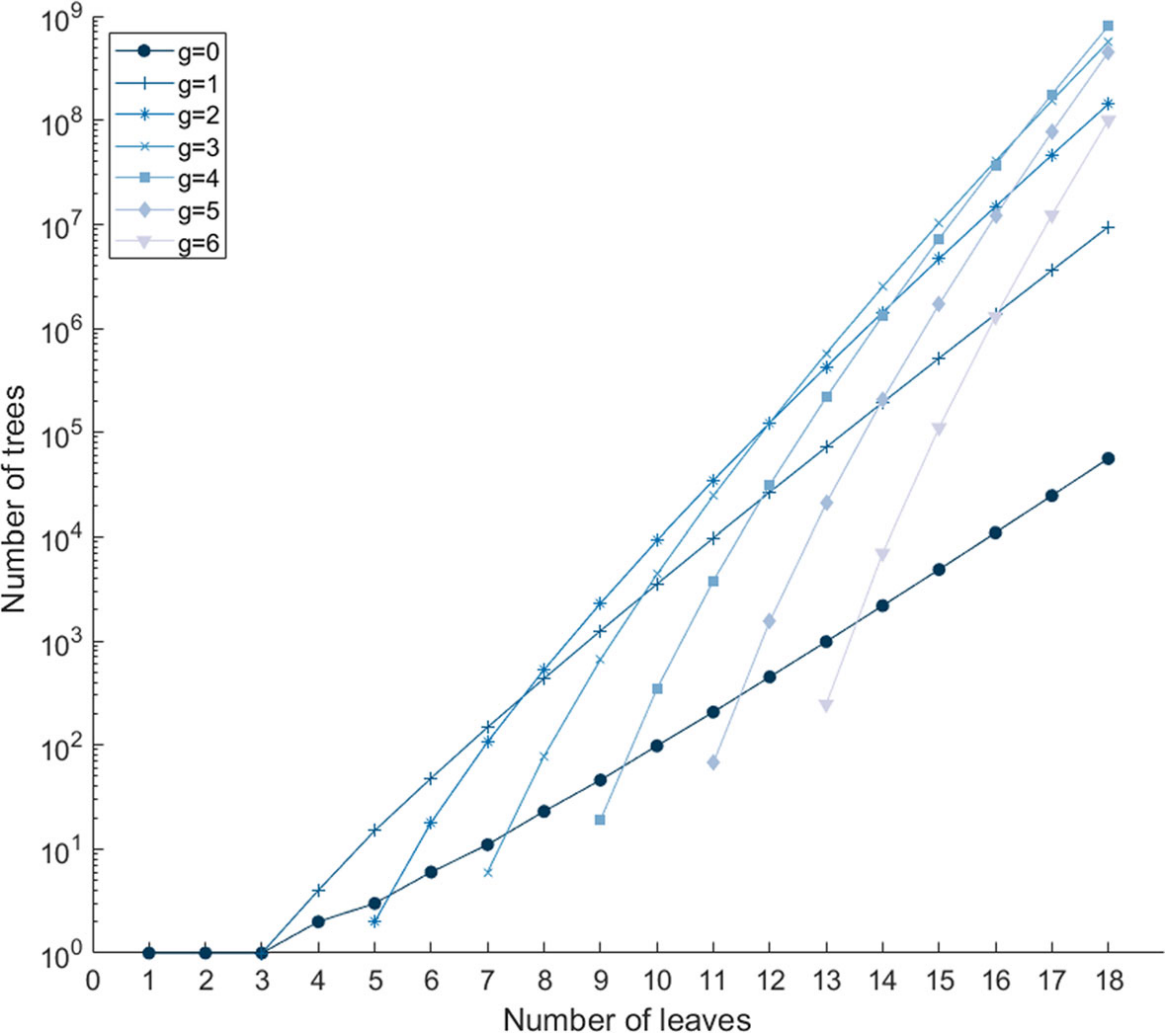
Number of galled trees as a function of the number of leaves *n*, for fixed numbers of galls $g=0, 1, 2, 3, 4, 5, 6$. Values are computed from Eqs. 26 and 27. The y-axis appears on a logarithmic scale

Figure 4 examines the growth of $E_{n,g}$ on a logarithmic scale for different fixed values of *g*. The number of trees with no galls has exponential growth $d_0 \rho ^{-n} n^{-\frac{3}{2}}$, for constants $d_0 \approx 0.3188$ and $1/\rho \approx 2.4833$ (Sect. 2.3). With one gall, $E_{n,1}$ exceeds $U_n$ with growth $d_1 \rho ^{-n} n^{\frac{1}{2}}$ for $d_1 \approx 0.3910$, but with the same exponential growth (Sect. 5.4). With specified numbers of galls $g \geqslant 2$, we see that growth of $E_{n,g}$ for fixed *g* appears to also follow an exponential trend.

**Fig. 5:**
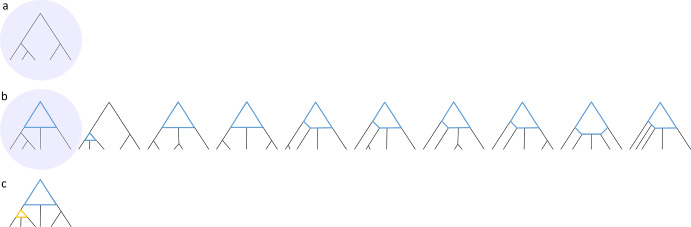
The addition of a gall to a galled tree. **A** One of the galled trees with 5 leaves and 0 galls. **B** The ten galled trees with 5 leaves and 1 gall produced by adding a gall to the tree in (A). The gall is indicated in blue. **C** The sole galled tree with 5 leaves and 2 galls produced by adding a gall to the tree highlighted in (B). The new gall is indicated in orange. A tree with 5 leaves has at most 2 galls (Color figure online)

## Discussion

Building on the Wedderburn-Etherington recursion for enumerating rooted binary unlabeled trees with *n* leaves (Eq. 1), we have introduced a recursion to enumerate rooted binary unlabeled (normal) *galled* trees with *n* leaves. The recursion follows the spirit of the Wedderburn-Etherington formula in its recursive descent from the tree root—but with additional terms for cases in which the root of the tree is also the top node of a gall (Eqs. 15 and 16). Continuing with a similar recursive strategy, we have also obtained a recursive formula for the number of galled trees with a fixed number of leaves *n* *and* a fixed number of galls *g* (Eqs. 26 and 27). We have derived generating functions for the number of galled trees (Eq. 36) and for the number of galled trees with 1 gall (Eq. 48), analyzing their asymptotic behavior.

Our numerical calculations find that for small *n*, for a fixed number of galls *g*, the increase of the number of galled trees $E_{n,g}$ with *n* appears faster for larger values of the fixed number of galls *g* (Table 3, Fig. 4). Because $E_{n,g}=0$ for $n<2g+1$, for higher values of *g*, values of $E_{n,g}$ at small *n* do not reflect the asymptotic trend. Nevertheless, for $g=1$, the initial apparent rapid growth of $E_{n,g}$ visible with increasing *n* moderates, in accord with the finding that the exponential order of the increase is the same as for the case of no galls (Sect. 5.4). A similar moderation in growth with increasing *n* is just observable for $E_{n,2}$, which could hint at a similar exponential growth; we can conjecture that each $E_{n,g}$ with fixed *g* has the same exponential growth. Note that Fuchs et al. (2019, Theorem 5.1; 2022, Theorem 1 and Corollary 2) showed that the exponential growth of labeled tree-child networks and normal networks with a fixed number of reticulation vertices (corresponding to a fixed number of galls in our case) is the same for any such number; only the subexponential growth differs.

On the other hand, when *g* is not restricted, we have shown in Sect. 5.2 that the convergence radius of the generating function ${\mathcal {A}}(t)$ satisfies $0<\alpha<\rho <1$, so that $A_n$ grows with $0.0779(4.8230^n)n^{-\frac{3}{2}}$. We also observed that the number of galled trees $A_n$ grows numerically faster with *n* than does the number of trees with no galls (Table 3).

For a fixed number of leaves *n*, the number of trees $E_{n,g}$ with a fixed number of galls increases to a maximum when the number of galls is at or near half the maximum number of galls $\lfloor \frac{n-1}{2} \rfloor$, then decreases. This pattern accords with the normal distribution we expect as *n* increases (Sect. 5.6). It is explained by the fact that many ways often exist to add a gall to a tree with a small number of galls without changing the number of lineages *n* (Fig. 5A). As the number of galls grows, fewer places are available in the tree to add more galls (Fig. 5B), and the number of possible trees declines. Informally, for a tree with *n* leaves, when we have a maximum of $g_{\max }$ potential galls from which to choose, the binomial $$\left( {\begin{array}{c}g_{\max }\\ g\end{array}}\right) $$, describing the number of possible subsets containing *g* galls, is highest for *g* near ${g_{\max }}/{2}$.

Galled trees provide a class of networks for use with biological processes such as admixture of populations, horizontal gene transfer, hybridization, and the recombination processes for which galled trees were originally introduced (Wang et al. 2001; Gusfield et al. 2004a). Other definitions of galled trees have previously been considered in enumerative problems (Semple and Steel 2006; Chang et al. 2018; Bouvel et al. 2020; Cardona and Zhang 2020); our definition, which requires galled trees to be “normal” by imposing a minimum of four nodes per gall, is designed for scenarios in which two lineages merge to form a new third lineage, but continue to have other descendants that are not descended from this merging event. Such scenarios are suited to phenomena such as admixture and hybridization, in which the merging process of two groups to form a third group has this feature: it does not cause the disappearance of the original two groups, which are free to produce additional descendants through processes that do not involve admixture and hybridization.

In related work, Cardona and Zhang (2020) enumerated rooted binary *labeled* normal galled trees. Their Theorem 8 finds that the number $M_n$ of such trees with *n* leaves is

58 $$\begin{aligned} M_n = \sum _{(k_2, k_3, \ldots , k_n) \in C} \frac{(n+k_2+\cdots +k_n-1)! \, 1^{k_3} \, 2^{k_4} \cdots (n-2)^{k_n}}{k_2! \, k_3! \, \cdots k_n! \, 2^{k_2+k_3+\cdots +k_n}}, \end{aligned}$$

where *C* is the set of vectors $(k_2,k_3,\dots , k_n)$ of nonnegative integers satisfying $1+k_2+2k_3+\dots +(n-1)k_n = n$. This enumeration accords with our enumeration of the corresponding unlabeled normal galled trees. For $n=1$ and 2, Eq. 58 produces 1 rooted binary labeled normal galled tree; for $n=3$, it gives 6 labeled trees—in accord with our count of 2 unlabeled normal galled trees, each of which has 3 possible labelings. For $n=4$, Eq. 58 gives 69 labeled trees; the 6 unlabeled normal galled trees in Table 2 have 12, 12, 3, 12, 6, and 24 labelings, respectively, summing to 69.

The enumeration of galled trees can assist in studies involving mixture processes in the same way that the Wedderburn-Etherington enumeration assists in evolutionary biology more generally, by describing the contents of a space of biologically relevant trees that must be traversed in a variety of algorithmic, combinatorial, probabilistic, and statistical problems [e.g. Harding (1971), Matsen and Evans (2012), Sievers et al. (2014), Colijn and Plazzotta (2018), Rosenberg (2021)]. The study adds to the growing area of enumerative combinatorics of phylogenetic networks [e.g. Bouvel et al. (2020), Cardona and Zhang (2020), Gunawan et al. (2020), Bienvenu et al. (2022), Fuchs et al. (2022)] and is one of relatively few studies to examine a class of unlabeled networks (Chang et al. 2018; Mathur and Rosenberg 2023). Further work can investigate the properties of $E_{n,g}$ for fixed $g \geqslant 2$.

## Appendix

This appendix shows that the radius of convergence $\alpha$ for the generating function ${\mathcal {A}}(t) = \sum _{n \geqslant 0} A_n t^n$ is positive. The approach is to bound $A_n$ from above by a quantity whose generating function is known to have a positive radius of convergence. For clarity in comparing with Bouvel et al. (2020) and Cardona and Zhang (2020), we retain the terms “rooted,” “labeled” and “unlabeled,” and “normal” in this appendix.

Our number of rooted unlabeled normal galled trees $A_n$ is bounded above by $M_n$, the corresponding number of rooted labeled normal galled trees in Theorem 8 of Cardona and Zhang (2020) (Eq. 58). $M_n$ is in turn bounded above by the number of rooted labeled galled trees tabulated without imposing the normality requirement, a quantity of Bouvel et al. (2020) that we call $Q_n$. Section 5 of Bouvel et al. (2020) showed that the exponential generating function ${\mathcal {Q}}(t) = \sum _{n \geqslant 0} Q_n t^n / n!$ has positive radius of convergence $r = \frac{1}{8}$.

To prove that $\alpha > 0$, we note that the number of rooted labeled normal galled trees with *n* leaves is $M_n = \sum _{i=1}^{A_n} M(T_i)$. Here, the sum proceeds over the $A_n$ rooted unlabeled normal galled trees, as each rooted labeled normal galled tree is obtained by placing a labeling on one of the rooted unlabeled normal galled trees. $M(T_i)$ is the number of labelings of rooted unlabeled normal galled tree $T_i$.

Next, consider the concept of a *symmetric node* of a rooted unlabeled galled tree, an internal node with two identical rooted unlabeled subtrees. The top node of a gall can be symmetric, but side nodes of a gall cannot, as one subtree of a side node contains the reticulation node and the other does not. A reticulation node also cannot be a symmetric node, as it has only one subtree.

For a rooted unlabeled normal galled tree, the number of distinct labelings is $L(T_i) = n! / 2^{s_i}$, where $s_i$ is the number of symmetric nodes of $T_i$. To see why this result holds, consider a planar representation of $T_i$, and examine all *n*! labelings of the *n* nodes. For each such labeling, for each symmetric node, a rotation of $T_i$ around the node generates a distinct labeling for the same labeled tree—so that each rooted labeled normal galled tree is obtained from $2^{s_i}$ of the *n*! labelings.

The number of symmetric nodes is bounded above by the maximal number of internal nodes that are not side nodes or hybridization nodes. This number is $n-1$, the number of internal nodes of a rooted tree with no galls; note that each gall adds two internal nodes to the tree, but neither of the “extra” nodes can be symmetric, as they include a side node and a reticulation node.

It is convenient to use *n* rather than $n-1$ for the upper bound on the number of symmetric nodes. Then

$$\begin{aligned} \frac{A_n}{2^n}n! = \sum _{i=1}^{A_n} \frac{n!}{2^n} < \sum _{i=1}^{A_n} \frac{n!}{2^{s_i}} = M_n \leqslant Q_n, \end{aligned}$$

so that

59 $$\begin{aligned} A_n < \frac{Q_n}{n!}2^n. \end{aligned}$$

The generating function ${\mathcal {Q}}(t)$ has positive radius of convergence $r=\frac{1}{8}$, so that ${\mathcal {Q}}(2t)$ converges for $|t| < \frac{1}{16}$. Multiplying Eq. 59 by $t^n$ and summing over all *n*, we have $|{\mathcal {A}}(t)| < | {\mathcal {Q}}(2t) |$ for $0< |t| < \frac{1}{16}$. Hence, the smaller ${\mathcal {A}}(t)$ must have positive radius of convergence $\alpha \geqslant \frac{1}{16}$. In particular, $\alpha >0$.

## References

[CR1] Bienvenu F, Lambert A, Steel M (2022). Combinatorial and stochastic properties of ranked tree-child networks. Random Struct Algorithms.

[CR2] Bouvel M, Gambette P, Mansouri M (2020). Counting phylogenetic networks of level 1 and 2. J Math Biol.

[CR3] Cardona G, Zhang L (2020). Counting and enumerating tree-child networks and their subclasses. J Comput Syst Sci.

[CR4] Chang K-Y, Hon W-K, Thankachan SV (2018) Compact encoding for galled-trees and its applications. In: 2018 Data Compression Conference, Snowbird, UT, pp 297–306

[CR5] Colijn C, Plazzotta G (2018). A metric on phylogenetic tree shapes. Syst Biol.

[CR6] Comtet L (1974). Advanced combinatorics.

[CR7] Drmota M (2009). Random trees.

[CR8] Felsenstein J (2004). Inferring phylogenies.

[CR9] Flajolet P, Sedgewick R (2009). Analytic combinatorics.

[CR10] Fuchs M, Gittenberger B, Mansouri M (2019). Counting phylogenetic networks with few reticulation vertices: tree-child and normal networks. Australas J Comb.

[CR11] Fuchs M, Huang E-Y, Yu G-R (2022). Counting phylogenetic networks with few reticulation vertices: a second approach. Discr Appl Math.

[CR12] Gascuel O (2005). Mathematics of evolution and phylogeny.

[CR13] Gunawan AD, Rathin J, Zhang L (2020). Counting and enumerating galled networks. Discrete Appl Math.

[CR14] Gusfield D (2005). Optimal, efficient reconstruction of root-unknown phylogenetic networks with constrained and structured recombination. J Comput Syst Sci.

[CR15] Gusfield D (2014). ReCombinatorics.

[CR16] Gusfield D, Eddhu S, Langley C (2003) Efficient reconstruction of phylogenetic networks with constrained recombination. In: Computational Systems Bioinformatics, CSB2003. Proceedings of the 2003 IEEE Bioinformatics Conference, Stanford, CA, pp 363–374 16452812

[CR17] Gusfield D, Eddhu S, Langley C (2004a) The fine structure of galls in phylogenetic networks. INFORMS J Comput 16:459–469

[CR18] Gusfield D, Eddhu S, Langley C (2004b) Optimal, efficient reconstruction of phylogenetic networks with constrained recombination. J Bioinform Comput Biol 2:173–213 10.1142/s0219720004000521 15272438

[CR19] Harding EF (1971). The probabilities of rooted tree-shapes generated by random bifurcation. Adv Appl Prob.

[CR20] Huson DH, Rupp R, Scornavacca C (2010). Phylogenetic networks: concepts, algorithms and applications.

[CR21] Kong S, Pons JC, Kubatko L, Wicke K (2022). Classes of explicit phylogenetic networks and their biological and mathematical significance. J Math Biol.

[CR22] Landau BV (1977). An asymptotic expansion for the Wedderburn–Etherington sequence. Mathematika.

[CR23] Mathur S, Rosenberg NA (2023). All galls are divided into three or more parts: recursive enumeration of labeled histories for galled trees. Algorithms Mol Biol.

[CR24] Matsen FA, Evans SN (2012). Ubiquity of synonymity: almost all large binary trees are not uniquely identified by their spectra or their immanantal polynomials. Algorithms Mol Biol.

[CR25] Meir A, Moon JW (1989). On an asymptotic method in enumeration. J Comb Theory Ser A.

[CR26] Meir A, Moon JW (1989). Erratum: on an asymptotic method in enumeration. J Comb Theory Ser A.

[CR27] Rosenberg NA (2021). On the Colijn–Plazzotta numbering scheme for unlabaled binary rooted trees. Discrete Appl Math.

[CR28] Semple C, Steel M (2003). Phylogentics.

[CR29] Semple C, Steel M (2006). Unicyclic networks: compatibility and enumeration. IEEE/ACM Trans Comput Biol Bioinform.

[CR30] Sievers F, Hughes GM, Higgins DG (2014). Systematic exploration of guide-tree topology effects for small protein alignments. BMC Bioinform.

[CR31] Song YS (2006). A concise necessary and sufficient condition for the existence of a galled-tree. IEEE/ACM Trans Comput Biol Bioinform.

[CR32] Steel M (2016). Phylogeny: discrete and random processes in evolution.

[CR33] Wang L, Zhang K, Zhang L (2001). Perfect phylogenetic networks with recombination. J Comput Biol.

[CR34] Warnow T (2018). Computational phylogenetics.

